# Alterations in immunophenotype and metabolic profile of mononuclear cells during follow up in children with multisystem inflammatory syndrome (MIS-C)

**DOI:** 10.3389/fimmu.2023.1157702

**Published:** 2023-04-20

**Authors:** Andreja Nataša Kopitar, Jernej Repas, Larisa Janžič, Maša Bizjak, Tina Tajnšek Vesel, Nina Emeršič, Mojca Zajc Avramovič, Alojz Ihan, Tadej Avčin, Mojca Pavlin

**Affiliations:** ^1^ Institute of Microbiology and Immunology, Faculty of Medicine, University of Ljubljana, Ljubljana, Slovenia; ^2^ Institute of Biophysics, Faculty of Medicine, University of Ljubljana, Ljubljana, Slovenia; ^3^ Department for Allergology, Rheumatology and Clinical Immunology, Children’s Hospital, University Medical Centre Ljubljana, Ljubljana, Slovenia; ^4^ Faculty of Medicine, Department of Pediatrics, University of Ljubljana, Ljubljana, Slovenia; ^5^ Group for Nano and Biotechnological Applications, Faculty of Electrical Engineering, University of Ljubljana, Ljubljana, Slovenia

**Keywords:** MIS-C, biomarkers, metabolism, B lymphocytes, lymphocyte activation, monocytes, dendritic cells, T lymphocytes exhaustion

## Abstract

**Introduction:**

Although children seem to be less susceptible to COVID-19, some of them develop a rare but serious hyperinflammatory condition called multisystem inflammatory syndrome in children (MIS-C). While several studies describe the clinical conditions of acute MIS-C, the status of convalescent patients in the months after acute MIS-C is still unclear, especially the question of persistence of changes in the specific subpopulations of immune cells in the convalescent phase of the disease.

**Methods:**

We therefore analyzed peripheral blood of 14 children with MIS-C at the onset of the disease (acute phase) and 2 to 6 months after disease onset (post-acute convalescent phase) for lymphocyte subsets and antigen-presenting cell (APC) phenotype. The results were compared with six healthy age-matched controls.

**Results:**

All major lymphocyte populations (B cells, CD4 + and CD8+ T cells, and NK cells) were decreased in the acute phase and normalized in the convalescent phase. T cell activation was increased in the acute phase, followed by an increased proportion of γ/δ-double-negative T cells (γ/δ DN Ts) in the convalescent phase. B cell differentiation was impaired in the acute phase with a decreased proportion of CD21 expressing, activated/memory, and class-switched memory B cells, which normalized in the convalescent phase. The proportion of plasmacytoid dendritic cells, conventional type 2 dendritic cells, and classical monocytes were decreased, while the proportion of conventional type 1 dendritic cells was increased in the acute phase. Importantly the population of plasmacytoid dendritic cells remained decreased in the convalescent phase, while other APC populations normalized. Immunometabolic analysis of peripheral blood mononuclear cells (PBMCs) in the convalescent MIS-C showed comparable mitochondrial respiration and glycolysis rates to healthy controls.

**Conclusions:**

While both immunophenotyping and immunometabolic analyzes showed that immune cells in the convalescent MIS-C phase normalized in many parameters, we found lower percentage of plasmablasts, lower expression of T cell co-receptors (CD3, CD4, and CD8), an increased percentage of γ/δ DN Ts and increased metabolic activity of CD3/CD28-stimulated T cells. Overall, the results suggest that inflammation persists for months after the onset of MIS-C, with significant alterations in some immune system parameters, which may also impair immune defense against viral infections.

## 1 Introduction

Multisystem inflammatory syndrome is a rare hyperinflammatory complication of SARS-CoV-2 infection in children and adolescents caused by severe immune activation (1, 2). Understanding of the distorted immune response and the time scale of its resolution in the convalescent phase is so far very limited. SARS-CoV-2 infection in children rarely progresses to severe disease (3, 4), however, increased number of adolescents and children with a rare, severe, and delayed inflammatory response were observed four to six weeks after mild or asymptomatic SARS-CoV-2 infection. This condition has been termed “multisystem inflammatory syndrome in children” (MIS-C) (5–9). Characteristic clinical features include high fever, skin and mucosal changes, gastrointestinal symptoms, and cardiac involvement, mainly myocarditis, sometimes with heart failure (5, 10).

The development of MIS-C is characterized by an uncontrolled immune response with hypersecretion of proinflammatory cytokines during the immune response to the virus (4, 5). Whole genome sequencing results suggest that a genetic predisposition contributes to the development of MIS -C, but further studies are needed to confirm this (11–13). Acute MIS-C was characterized by high systemic levels of inflammatory cytokines such as interleukin-1β (IL-1β), IL-6, IL-8, IL-10, IL-17, and IFN-γ (14), as well as increased levels of circulating spike protein and NF- κB dependent response (12). In addition, a cytokine profile indicating mucosal recruitment of NK cells, T cells, monocytes, and neutrophils, and regulation of the adaptive immune system through a negative feedback loop has been reported (7, 15). It was also suggested that the superantigenic nature of the SARS-CoV-2 spike protein (16, 17), together with its long-term persistence in some tissues and compromised gut mucosal barrier leading to chronic activation and hyperinflammation (8) could partially explain the development of MIS-C. Despite the relatively well-characterized immune changes in acute MIS-C, much less is known about their persistence in convalescent MIS-C patients in the convalescent (post-acute) phase (PA). Various (e.g., genetic) parameters and markers associated with MIS-C (11) were studied, and a detailed longitudinal study of immunopathologic signatures has recently been published (12). However, many questions regarding changes in immune cell populations of PBMCs in convalescent MIS-C patients have not yet been investigated.

Additionally, immunometabolism has emerged as a field interconnecting cell energy metabolism with the immune cell differentiation and functions. Studies have shown substantial changes in the metabolic profile of immune cells during coronavirus disease (COVID-19), including mitochondrial dysfunction in live peripheral blood mononuclear cells (PBMCs) with increased rate of glycolysis (18), altered metabolic profile of monocytes with impaired oxidative burst (19), and T cell exhaustion and senescence (20). However, very little is known about the metabolic profile of immune cells in MIS-C, particularly in the convalescent phase.

To this end, we performed detailed flow cytometric immunophenotyping of various lymphocyte subsets and antigen-presenting cells (APCs) in the peripheral blood of MIS-C patients in the acute phase and in the convalescent phase after two to six months. Results were compared with age-matched healthy controls. Our aim was to determine the extent to which the characteristics and mechanisms of the activated/exhausted immune response persist. To further analyze any potential alterations in the convalescent MIS-C phase, we performed detailed immunometabolic profiling of PBMCs in the convalescent phase.

## 2 Materials and methods

### 2.1 Study design

Fourteen children with MIS-C and an average age of 10.9 years were enrolled at the University Children’s Hospital of Ljubljana, Department of Allergology, Rheumatology, and Clinical Immunology. Among all patients, 14 patients were selected who had already been examined in the acute and convalescent phases by the end of the study. The MIS-C diagnosis was based on the United States Centers for Disease Control and Prevention (CDC) and World Health Organization (WHO) case definition (21, 22). Blood samples of MIS-C patients were collected at admission to the hospital before treatment. Previous exposure to SARS-CoV-2 was determined by PCR and antibody measurement. Blood samples were collected again from the same patients 2-6 months (median 3.2 months) after the onset of fever. Blood samples were also collected from six age-matched healthy controls. None of the healthy controls had a history of COVID-19 or a contact with a SARS-CoV-2 positive person prior to blood sampling and all had negative SARS-CoV-2 antibodies. The study protocol was approved by the National Ethics Committee of Slovenia under registration number 0120-211/202/7. Up to 8 ml of blood was collected in BD Vacutainer heparin tubes and immediately processed for detection of T and B cells differentiation and APCs phenotype. Samples from the convalescent MIS-C patients were divided into two parts, one for immunophenotyping (flow cytometry) and one for immunometabolic profile measurements (Seahorse XFe24 Analyzer). For the latter, PBMCs were isolated from blood using Ficoll Paque PREMIUM 1.073 according to a standard isolation protocol.

### 2.2 Lymphocyte subpopulations

Quantification and enumeration of major lymphocyte subsets was performed by labeling 50 μl of fresh blood samples with 10 μL of BD Multitest 6 color TBNK monoclonal antibodies mixture (contents: CD3 FITC/CD16 PE + CD56 PE/CD45 PerCP-Cy5.5/CD4 PE -Cy7/CD19 APC/CD8 APC-Cy7) (BD Bioscience San Josè, CA, USA), according to the manufacturer’s procedure. BD Trucount™ tubes were used to identify and determine the percentage and absolute number of T, B, and NK cells, as well as CD4 and CD8 subpopulations of T cells. Cells were measured and analyzed using the BD FACSCanto™ II flow cytometer and BD FACSCanto v 3.0 software. The laboratory report of the major lymphocyte populations in the BD FACSCanto v 3.0 software is shown in **Supplementary Figure S1**.

### 2.3 T cell Immunophenotyping

For detailed analysis of T cell phenotype, 100 μl of blood samples were stained with a cocktail of the following monoclonal antibodies (mAbs): CD3 PE-Cy7, CD31 APC (eBioscience, San Diego, CA, USA), CD4 V450, CD8 APCy7, HLA-DR PerCP-Cy5.5, TCRα/β FITC, TCRγ/δ PE, and CD45RA V500 (all from BD Bioscience) for 20 minutes at room temperature. Erythrocytes were lysed with BD FACS Lysing solution (BD Bioscience) and washed twice with PBS containing 1% BSC. At least 500,000 cells per sample were collected using a BD Canto II flow cytometer (BD Biosciences, San Jose, CA, USA). T cell subsets were classified into i) cytotoxic T cells, ii) helper T cells, iii) recent thymus emigrants (CD4+ CD45RA CD31+) T cells, iv) naive (CD45RA+CD4+) helper T cells, (v) memory helper T cells (CD45RO+CD4+), (vi) T cells with T cell receptor (TCR) alpha/beta and gamma/delta, (vii) double negative T cells (DN T- CD4-CD8-): with alpha/beta TCR and gamma/delta TCR, and viii) activated (HLA-DR +) T cells. The volume of antibodies per test are shown in **Supplementary Table S1**, and the gating strategy is shown in **Figure S2**. Samples were analyzed using FlowJo 10.8 and BD FACSDiva v8.0.1 software (TreeStar Inc.) (Becton Dickinson, San Josè, CA).

### 2.4 B cell differentiation

B cell differentiation in peripheral blood was detected in 100 μl of blood samples washed twice with staining buffer (PBS containing 1% BSA) and stained with a cocktail of mAbs: CD45 V500, CD19 PE-Cy7, CD21 APC, IgM FITC, IgD PE, CD27 PerCP-Cy5.5, and CD38 V450 (all from BD Bioscience) for 20 minutes in the dark at room temperature. Erythrocytes were lysed with BD FACS Lysing solution (BD Bioscience) and washed twice with buffer. B cells were then classified according to their maturation stage into (i) immature (CD21-CD27-) B cells, (ii) naive (CD21+CD27-IgM+) B cells, (iii) memory B cells (CD27+CD19+), (iv) unswitched memory B cells (CD27+IgM+IgD+), (v) memory-switched B cells (CD27+IgM-IgD-), (vi) transitional B cells (CD38+IgM+), and (vii) plasmablasts (CD38+IgM-) (23) For gating strategy see **Supplementary Figure S3**, volumes of antibodies per test are shown in **Table S1**. At least 500,000 cells per sample were collected using a BD Canto II flow cytometer. Data were analyzed using FlowJo 10.8 and BD FACSDiva v8.0.1 software (TreeStar Inc.) (BD Biosciences, San Jose, CA, USA).

### 2.5 APC immunophenotyping

APCs from peripheral blood were analyzed in 100 μl blood samples. Cells were stained with mAb cocktail for 30 minutes at room temperature. Ab/test volumes and other data for antbodies are described in **Table S1**. Erythrocytes were lysed with BD FACS Lysing solution (BD Bioscience) and washed twice with buffer. Congenital APCs were identified by expression of the surface markers HLA-DR APC -Cy7, CD14 PerCP-Cy5.5, CD16 PE (BD Biosciences) (classical monocyte CD14+CD16-), CD303 FITC, CD123 PE -Cy7 (plasmacytoid DCs), CD11c APC, CD141 ViolB (Miltenyi Biotec, Bergisch Gladbach, Germany) (conventional DC1s), CD1c V500 (BD Pharmingen, San Diego, USA) (conventional DC1s). Flow cytometric analyzes were performed using FACS Diva software (BD Biosciences, San Jose, CA, USA) on and BD Canto II. Data were analyzed using FlowJo software version 10 (Tree Star). The APC immunophenotyping gating strategy is shown in **Supplemental Figures S4**, **S5**.

### 2.6 Mitochondrial bioenergetics and metabolic assays

A detailed analysis of immunometabolism of isolated PBMCs was performed. PBMCs isolated from blood of convalescent MIS-C patients and healthy controls (N = 6) were separated into four groups: PBMCs (unstimulated PBMCs), PMA/ionomycin-stimulated PBMCs (PMA (phorbol-12-myristate-13-acetate)/ionomycin PBMCs), PBMCs for separation of monocytes and lymphocytes, and PBMCs for activation with CD3/CD28 antibodies (aCD3/aCD28 PBMCs). The metabolic profile and mitochondrial function of PBMCs, PMA/ionomycin activated PBMCs, separated lymphocytes, monocytes, and aCD3/aCD28 activated PBMCs were determined with XFe24 Seahorse analyzer using Mito Stress Assay (Agilent, USA) with the addition of fourth injection of PMA/ionomycin to measure oxidative burst.

Monocytes were isolated from PBMCs by negative magnetic separation using MojoSort™ Nanobeads (Biolegend) according to the manufacturer’s instructions. The cells remaining after magnetic separation were designated as lymphocytes. A small fraction of cells from all sample groups was measured by flow cytometry to determine the percentage of specific cell fractions (monocytes, lymphocytes using mAb CD14 PerCP-Cy5.5 and CD3 V450, both from BD Bioscience).

#### 2.6.1 Determination of immunometabolic profile and mitochondrial function

For PMA/ionomycin-stimulated PBMCs, PBMCs were seeded on a Seahorse cell plate immediately following isolation in triplicate at a concentration of 2.5-4×10^5^ cells/well and incubated for 4 h at 37°, 5% CO2 with 100 ng/mL PMA (Sigma-Aldrich, USA) and 1.0 μg/mL ionomycin (Sigma-Aldrich, USA). All other cell types were seeded on Seahorse plate 4 hours after PBMC isolation. Unstimulated PBMCs were seeded in triplicate at a concentration of 2.5-4×10^5^ cells/well. Monocytes isolated by magnetic separation were seeded in duplicates at a concentration of at least 0.5-1.5 × 10^5^ cells/well. Because of the limited amount of blood, monocytes were measured in only a single well in some samples. The remaining cells, which consisted mainly of lymphocytes (referred to as lymphocytes), were seeded in triplicate at a concentration of 2-3×10^5^ cells/well. At 4 hours after isolation, PBMCs, PMA/ionomycin-stimulated PBMCs, monocytes, and lymphocytes were measured using the Seahorse XFe24 analyzer according to the standard Mito Stress Test protocol.

#### 2.6.2 Activation of PBMCs with aCD3/aCD28

Part of PBMCs were seeded on 24-well plate at 1×10^6^ cells/ml and stimulated for 48 hours with anti-CD3 (1 μg/mL) plus anti-CD28 (5 μg/mL) (both Invitrogen, Thermo Fisher Scientific) in RPMI + 10% FBS media (Sigma-Aldrich). After 48 hours of stimulation, the cells were counted and seeded in triplicate at a concentration of 1.5-2×10^5^ cells/well on Seahorse plates. Seahorse measurements of CD3/CD28-activated PBMCs were performed 48 hours after isolation of PBMCs.

#### 2.6.3 Seahorse analyzer measurements

Real-time measurements of OCR (oxygen consumption rate) and ECAR (extracellular acidification rate) were performed with the XFe24 Seahorse Extracellular Flux Analyzer (Agilent, Santa Clara, CA, USA) using the Mito Stress kit according to the manufacturer’s instructions. Cells (PBMCs, PMA/ionomycin stimulated PBMCs, monocytes, lymphocytes) were centrifuged, resuspended in Seahorse XF RPMI 1640-based Seahorse XF Glycolytic Rate Assay Medium (5.6 mM glucose, 2 mM L-glutamine, 0 mM sodium pyruvate, 1 mM HEPES, equilibrated to pH 7.4) (Agilent) and plated on a 24-well Seahorse cell culture microtiter plates covered with CellTak^®^ with a specified number of cells at 0.1 mL per well. The plates were spun down at 200 g for 1 minute and incubated at 37 °C for 15 minutes without CO_2_. Seahorse XF medium (0.4 mL) was then added and the plate was incubated for an additional 30 minutes at 37 °C without CO_2_. The Seahorse Mito Stress Assay was performed using 1.5 μM oligomycin, 2 μM FCCP, and 0.5 μM rotenone/antimycin A (Agilent) for PBMCs, PMA/iono PBMCs, lymphocytes, and aCD3/aCD28 activated PBMCs. For monocytes, 2 μM oligomycin, 0.5 μM FCCP, and 0.5 μM rotenone/antimycin A were used. As a fourth injection, PMA and ionomycin were injected with a final concentration in the media of 100 ng/ml (PMA) and 1.0 μg/ml (ionomycin), respectively, which enabled us to determine the oxidative burst as maximal OCR at two time points after injection of PMA/iono. The maximal respiration (maximal OCR) that reflects the maximal capacity of mitochondria for oxidative phosphorylation, and spare mitochondrial capacity, were also determined according to Mito Stress Assay.

The parameters of the mitochondrial respiratory function were calculated from the OCR profile: basal OCR (before the addition of oligomycin injection), OxPhos ATP (OCR-linked ATP production rate; calculated from the difference between the basal OCR rate and the oligomycin-induced OCR rate: OxPhos ATP (pmol ATP/min) = OCR ATP (pmol O2/min) * 2 (pmol O/pmol O2) * P/O (pmol ATP/pmol O), maximal OCR (OCR after FCCP injection) and spare respiratory capacity (maximal OCR – basal OCR). Basal ECAR values were also measured and ATP production rate from glycolysis was calculated according to equation: glycoATP (pmol ATP/min) = glycoPER (pmol H+/min) = basalPER (pmol H+/min) – mitoPER (pmol H+/min) = basalPER – (basal OCR – OCR after rotenone/antimycin A) * 0.6. All measured parameters are presented normalized to the number of cells per well.

### 2.7 Statistical analysis

Statistical analyzes were performed with GraphPad Prism software (version 9.0, GraphPad Software Inc, La Jolla CA). All graphs were generated using GraphPad Prism software. All data were tested for normality using the Shapiro-Wilk test. Comparisons between acute MIS-C and convalescent values were assessed with the Wilcoxon test if the distribution was not normal and with the paired t-test if the data were normally distributed. Comparison between the patient groups and healthy controls was determined with the Mann-Whitney test when the distribution was not normal and with the unpaired t-test when the distribution was normal. P ≤ 0.05 was considered statistically significant. Spearman correlation analysis was used to provide additional insight into possible relationships between the clinical, immunologic, and metabolic parameters.

## 3 Results

### 3.1 Patient characteristics

Fourteen MIS-C patients and six healthy controls (HC) were included in the study. The mean age at diagnosis was 10.9 years (range 4.1 – 15.7 years), eight patients were male and six female. The mean age of the healthy control subjects was 10.8 years (range 7.5 – 13.7 years), five were female and one was male. None of the HC had a history of COVID-19 or contact with a SARS-CoV-2-positive person before blood collection, and all tested negative for SARS-CoV-2 antibodies. The clinical and laboratory data of the MIS-C patients during the acute MIS-C (A) are shown in **Table 1**. At admission, all MIS-C patients had positive SARS-CoV-2 serology and a negative SARS-CoV-2 PCR test from a nasal swab. All included MIS-C patients were treated with intravenous immunoglobulins and methylprednisolone, and three patients were additionally treated with the IL-1 receptor antagonist anakinra. Two patients required care in an intensive care unit (one of them was also in the group treated with IL-1 receptor antagonist).

**Table 1:** Detailed clinical and laboratory data of MIS-C patients in the acute phase (n=14).

Signs and symptoms		
Days with fever, median (IQR)		6 (4 – 7)
n of organ systems involved, n (%)	2-3	3 (21%)
	4-5	6 (43%)
	≥ 6	5 (36%)
**Organ system involvement**		**N (%)**
**Hematologic**		14 (100)
Elevated D-dimer		14 (100)
Thrombocytopenia		6 (43)
Lymphopenia		14 (100)
Hepato-/splenomegaly		7 (50)
**Dermatologic/mucocutaneous**		13 (93)
Rash		9 (64)
Conjunctivitis		6 (43)
Mucocutaneous lesions		6 (43)
**Gastrointestinal**		13 (93)
Abdominal pain		11 (79)
Vomiting		9 (64)
Diarrhea		8 (57)
**Cardiovascular** ^¶^		10 (71)
Elevated troponin		10 (71)
Myocarditis		8 (57)
Cardiac dysfunction		7 (50)
**Neurologic**		9 (64)
Headache		8 (57)
Encephalopathy		1 (7)
**Respiratory** ^§^		8 (57)
Cough		7 (50)
Chest pain		2 (14)
Renal**		1 (7)
Lymphadenopathy***		8 (57)
**Laboratory parameters**		**Median (IQR)**
CRP, peak (mg/l)		167 (91 – 210)
D-dimer, peak (µg/l)		3570 (1884 – 6438)
Troponin, peak (ng/l)		467 (23 – 801)
BNP, peak (pg/ml)		3363 (2157 – 12562)
Ferritin, peak (µg/l)		366 (210 – 709)
Haemoglobin, nadir (g/dl)		10.9 (10.3 – 11.3)
Lymphocytes, nadir (cells/µl)		705 (377 – 865)
Platelets, nadir (10^3^ cells/µl)		157 (106 – 190)

BNP, natriuretic peptide test; CRP, C-reactive protein; IQR, interquartile range; MIS-C, multisystem inflammatory syndrome in children.

^¶^Cardiovascular – also includes coronary artery dilation or aneurysm, hypotension, pericardial effusion, mitral regurgitation.

^§^Respiratory – also includes dyspnoea, pulmonary oedema, pneumonia, acute respiratory distress syndrome, pleural effusion.

**Renal – Acute kidney injury.

***Lymphadenopathy – cervical lymphadenopathy > 1.5 cm diameter, generalised lymphadenopathy, mesenterial lymphadenopathy.

Median time interval from SARS-CoV-2 infection to onset of MIS-C was 42 days (IQR 26 – 55 days) days and median time interval from fever onset to hospital admission was 4 days (IQR 3 – 5 days).

The median time interval between blood draws in the acute phase (A) and the convalescent (post-acute) phase (PA) was 3.7 months (IQR 2.2 – 5.8). Two patients were re-infected with SARS-CoV-2 1.8 and 3.4 months before blood sampling in the convalescent phase, respectively. In both cases, reinfection was mild with fever and upper respiratory tract symptoms, one patient also experienced headache and changes in sense of smell and taste. All laboratory parameters normalized in the convalescent phase, while clinically 10/14 (71%) patients showed complete resolution of symptoms and 4/14 (29%) patients continued to have mild symptoms such as fatigue, arthralgia and subfebrile temperature.

### 3.2 Lymphocytopenia is diminished in the convalescent phase of MIS-C patients

**Figure 1:**
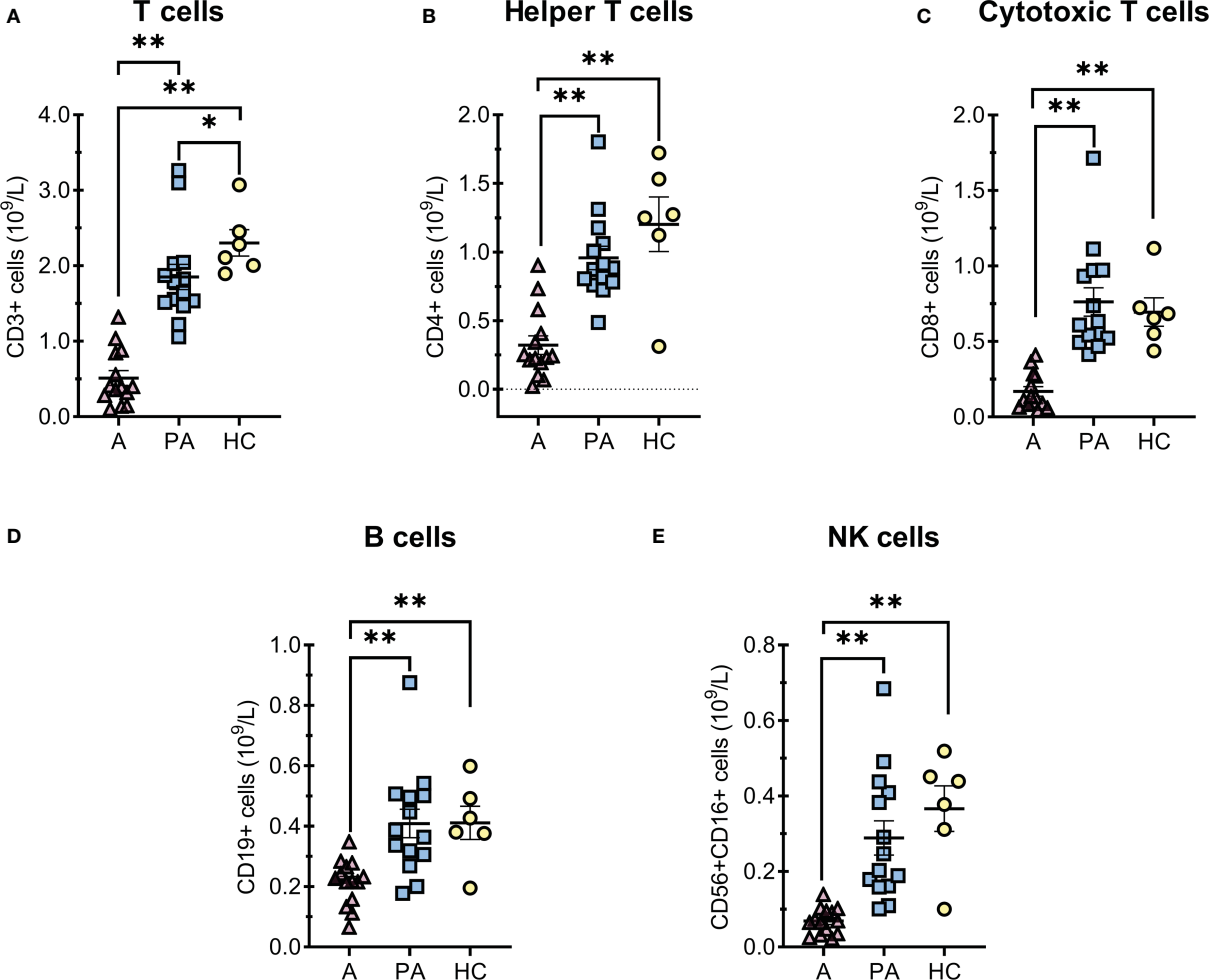
T and B cells alterations in MIS-C patients in the acute **(A)** and convalescent (post-acute) phase (PA) compared with age-matched healthy controls (HC). A-E, concentrations of **(A)** total T cells, **(B)** CD4+ helper T cells, **(C)** CD8+ cytotoxic T cells, **(D)** total B cells and **(E)** NK cells × 10^9^ per liter of blood. Individual value plots show a point for the actual value of each observation in a group. Pink triangles represent patients with acute MIS-C (A, n = 14), blue squares represent patients with convalescent MIS-C (PA, n = 14), and yellow dots represent healthy controls (HC, n = 6). Data are presented as means ± SEM and were tested for normal distribution using the Shapiro-Wilk normality test. Significance tests between patients by phase of illness (A – acute, PA - convalescent) were performed using the paired-samples t-test for normal distribution or the Wilcoxon test otherwise. For comparison between patient groups and HC, *P ≤ 0.05 and **P ≤ 0.01 were determined with an unpaired t-test if the distribution was normal and with the Mann-Whitney test otherwise.

Analysis of the lymphocyte subpopulations at disease onset (acute phase) revealed decreased concentrations of all major lymphocyte populations compared with the convalescent (post-acute) phase and healthy controls (**Figure 1**). Concentrations of most lymphocyte populations returned to normal in the convalescent phase with no observed differences versus healthy controls (**Figure 1**), except for total T-cells concentrations, which were still slightly lower compared to the control group (**Figure 1A**).

### 3.3 T cell Immunophenotyping

**Figure 2:**
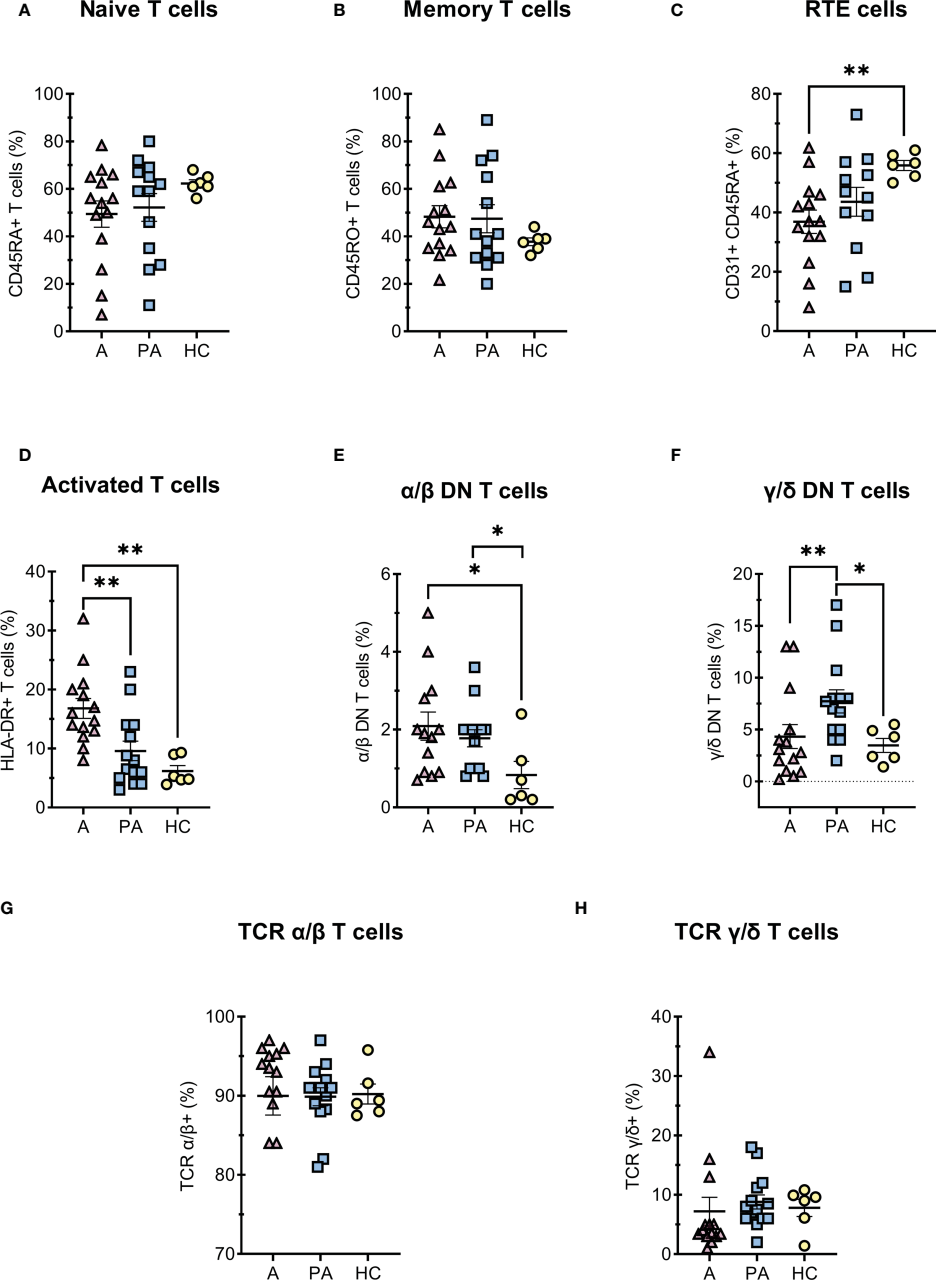
T cells diferentiation in MIS-C patients during acute and convalescent phase compared to age-matched HC. A-C, (percentages of CD4+ T cells) **(A)** naive CD45RA+ CD4+ T cells, **(B)** memory CD45RO+ CD4+ T cells, **(C)** RTE - recent thymic emigrants CD31+CD45RA+ CD4+ T cells; **(D)** (percentages of CD3+ T cells) activated HLA-DR+ T cells; E-F (percentages of CD3+, CD4- and CD8- T cells) **(E)** DN T α/β, **(F)** DN T γ/δ; G-H (percentages of CD3+ T cells) **(G)** α/β T cells and **(H)** γ/δ T cells. Pink and blue shading represents patients with MIS-C in acute (A, n = 12-14) or convalescent (PA, n = 13-14) phase of the illness. Yellow shading represents healthy controls (HC, n = 6). Data are presented as means ± SEM. The Shapiro-Wilk normality test was used to test for the normal distribution. Significance testing between patients by phase of illness (A – acute, PA – convalescent) was performed using paired t-test for normal distribution or Wilcoxon test otherwise. For comparison between patient groups and HC, *P ≤ 0.05 and **P ≤ 0.01 were determined by un-paired t-test if the distribution was normal and Mann-Whitney test otherwise.

In general, concentrations of all T cell subpopulation were markedly reduced in the acute phase (A) due to the profound lymphopenia of T cells (**Figure 1**). However, a similar trend was not always observed when the percentages of these cells were compared, for example in activated and DN T cells (**Figures 2D–F**). The percentage and concentration of RTE (Recent Thymic Emigrants - CD31+ CD45RA+) helper T cells, which reflect the current production of helper T cells in the thymus, were greatly reduced during acute MIS-C but the values normalized in convalescent MIS-C (PA) (**Figure 2C**).

Among T cells, we further analyzed double-negative T (DN T) cells, which lack expression of CD4 and CD8 co-receptors. DN T cells are divided into α/β-DN T and γ/δ-DN T subpopulations, depending on whether their TCR consists of α/β- or γ/δ-chains. The percentage of double-negative α/β-DN T cells was increased in the acute and convalescent MIS-C compared with healthy controls, whereas the percentage of γ/δ- DN T cells increased later in the convalescent MIS-C group (**Figures 2E, F**). Importantly, these cells are formed after strong and prolonged T cell activation, and represent one of the residual cell populations. On the other hand, we found no difference in the proportion of α/β or γ/δ T lymphocytes between the acute, convalescent MIS -C and healthy controls (**Figures 2G, H**). We also observed that T cells were strongly activated in the acute phase compared with HC (**Figure 2D**). Moreover, the median fluorescence intensity (MFI) of CD4 and CD8 co-receptors expression on T cells was significantly decreased in the acute phase compared with the convalescent phase and HC (**Figures S6B, C**). Although CD4 and CD8 expression normalized in the convalescent phase, CD3 expression remained significantly reduced compared with HC (**Figure S6A**).

### 3.4 B cell differentiation

**Figure 3:**
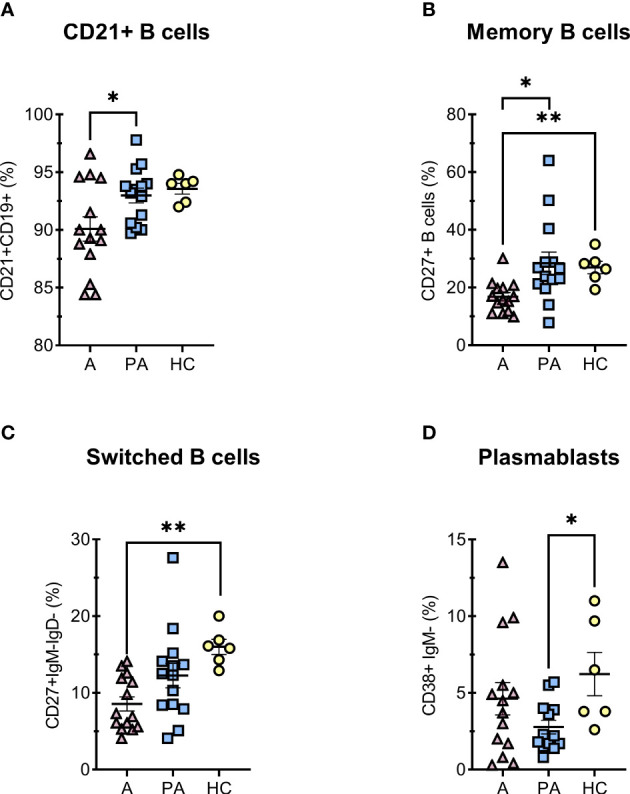
Percentage of B cell subpopulations in peripheral blood of MIS-C patients in acute **(A)** and convalescent (PA) phase compared with age-matched healthy controls (HC). A–F, percentages of (% of all CD19+) **(A)** immunocompetent mature B cells CD21+, **(B)** memory CD27+IgM- B cells, **(C)** memory-switched CD27+IgM-IgD- B cells and **(D)** plasmacytoid CD38+IgM- B cells. Pink and blue shading represents patients with MIS-C in acute (A, n = 14) and convalescent (PA, n = 14) phase of the illness. Yellow shading represents healthy controls (HC, n = 6). The Shapiro-Wilk normality test was used to test for the normal distribution. Data are presented as means ± SEM. Significance testing between patients by phase of illness (A – acute, PA – convalescent) was performed using paired t-test for normal distribution or Wilcoxon test for not normal distribution. For comparison between patient groups and HC, *P ≤ 0.05 and **P ≤ 0.01 were determined by using unpaired t-test, if the distribution was normal and Mann-Whitney test for not normal distribution.

Since many studies have reported dysregulations of B lymphocyte differentiation in COVID-19 (23–25), we also analyzed for different B cell populations (**Figures 3**, **S8**). In acute MIS-C, we observed a decreased proportion of functional B cells expressing the complement receptor CR2 (CD21) (**Figure 3A**). Compared with healthy controls, we also observed a marked reduction in memory B cells (CD27+CD19+) and memory-switched B cell (CD27+CD19+CD21+IgM-IgD-) in the acute phase, but the proportion of these cells normalized in convalescent phase (**Figures 3B, C**), while concentration of memory-switched cell remained decreased in convalescent MIS-C compared to HC (**Figure 3C**). The dysregulations in B cells differentiation characteristic of coronavirus infection, manifested by impaired isotype switching, were also reflected in a reduced proportion of plasmablasts in convalescent MIS-C compared with HC (**Figure 3D**).

### 3.5 APC immunophenotyping

**Figure 4:**
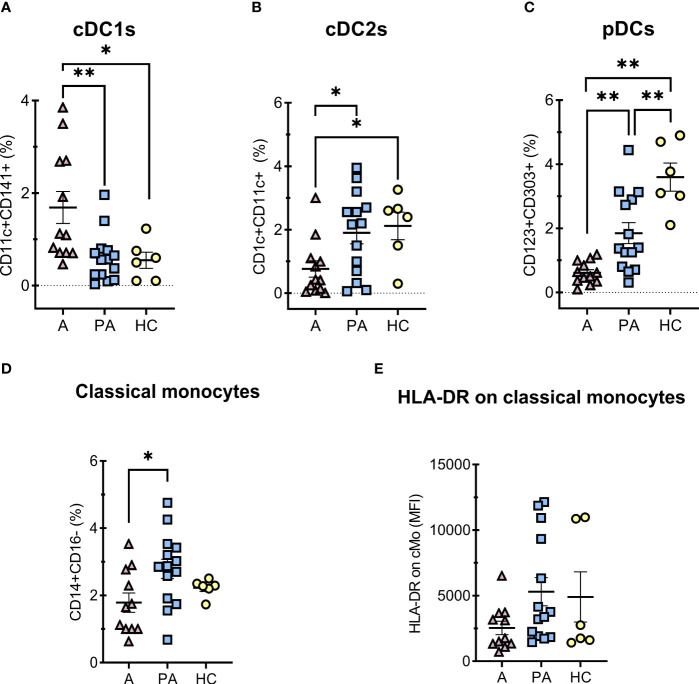
Percentages of DCs and classical monocytes in peripheral blood of MIS-C patients in acute **(A)** compared to convalescent phase (PA) and age-matched healthy controls (HC). A-C, percentages of PBMC, HLA-DR positive and CD14 negative cells **(A)** conventional type 1 dendritic cells (cDC1s) – CD11c+CD141+HLA-DR+CD14-, **(B)** conventional type 2 dendritic cells (cDC2s) – CD1c+CD11c+ HLA-DR+CD14-, **(C)** plasmacytoid dendritic cells (pDCs) – CD123+CD303+CD11c-HLA-DR+CD14-, **D**) classical (CD14+CD16-) monocytes percentages of HLA-DR positive vs SSC subset, and **E)** expression of HLA-DR (MFI) on classical monocytes. Pink and blue shading represents MIS-C patients in acute (A, n = 12) and convalescent (PA, n = 14) phase of the illness, respectively. Yellow shading represents HC (n = 6). The Shapiro Wilk test was used to test for normality. Significance testing between patients by phase of illness (A – acute, PA – convalescent) was performed using paired t-test for normal distribution or Wilcoxon test otherwise. For comparison between patient groups and HC, *P ≤ 0.05 and **P ≤ 0.01 were determined by using unpaired t-test if the distribution was normal and Mann-Whitney test otherwise. Data are presented as median with 95%CI.

Dendritic cells (DC) and monocytes from COVID-19 patients as well as from MIS-C patients have significantly impaired immune regulation (26, 27). Therefore, we analyzed different proportions of subpopulations of APCs (**Figure 4**). We observed a higher proportion of cDC1 in peripheral blood in the acute phase compared with the convalescent phase and healthy controls (**Figure 4A**). In contrast, a strong depletion of pDCs was observed in the acute phase compared with the convalescent phase and HC. The difference was still observed in the convalescent phase in MIS-C patients compared with HC, although to a lesser extent (**Figure 4C**). The percentage of cDC2s was lower in the acute phase compared to HC (**Figure 4B**).

The proportion of classical monocytes (CD14+CD16-) (28) was significantly lower in the acute phase of MIS-C than in the convalescent phase (**Figure 4D**). We also observed a tendency toward lower expression of HLA-DR on monocytes in the acute phase than in the convalescent phase, but the difference was not significant (**Figure 4E**).

### 3.6 Immunometabolism of isolated PBMCs – Seahorse assay

#### 3.6.1 Mitochondrial reprogramming in PBMCs of convalescent MIS-C patients

**Figure 5:**
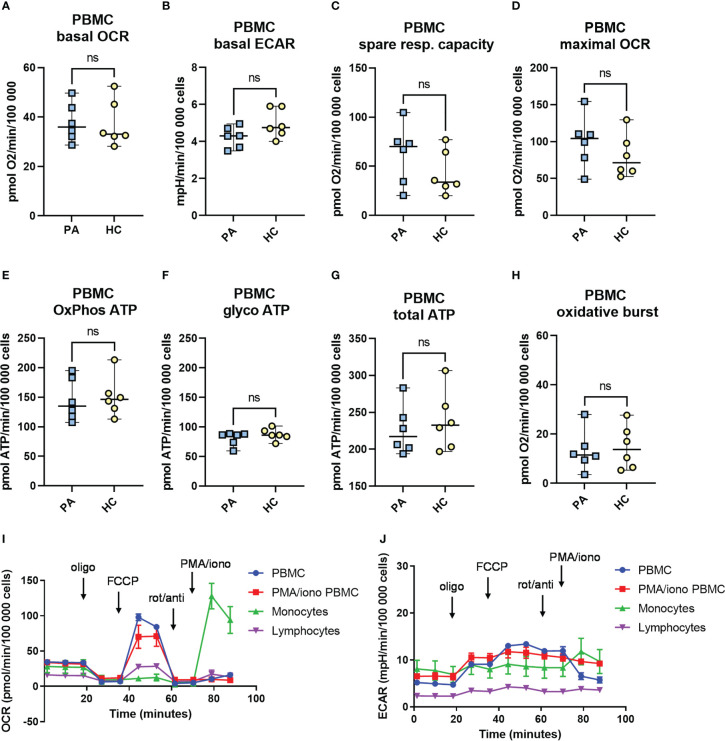
The metabolic profile and mitochondrial function in peripheral blood mononuclear cells (PBMCs) from patients with MIS-C in convalescent phase. Metabolic profile of PBMCs of healthy controls (HC, n = 6), and convalescent MIS-C patients (PA, n = 6) was determined with Seahorse XFe24 analyzer. PBMCs were isolated and seeded in a Seahorse plates. The oxygen consumption rate (OCR) **(A)** and extracellular acidification rate (ECAR) **(B)** were measured in real time under basal conditions, and in response to the injection of the following inhibitors: oligomycin (Oligo, 1.5 μM), cyanide‐4‐ (trifluoromethoxy) phenylhydrazone (FCCP, 2 μM), rotenone plus antimycin-A (rot/anti, 0.5 μM) and to PMA/ionomycin (PMA/Iono, 1 μg/ml) to measure oxidative burst. The spare mitochondrial capacity **(C)** and maximal respiration - maximal OCR **(D)** were determined according to Mito Stress Assay, OxPhos ATP **(E)**, glycoATP **(F)** and total ATP **(G)** were calculated from OCR and ECAR measurements as defined in M&M. The oxidative burst **(H)** was measured after last injection with PMA/ionomycin (1 μg/ml). Data are presented as median with 95%CI and analyzed by Mann-Whitney test ns, not significant. **(I, J)** Representative time-course measurements of OCR and ECAR and the injection strategy.

Immunometabolic profile of PBMCs was determined (**Figure 5**). No significant differences were observed between the convalescent MIS-C and healthy controls in any of the analyzed parameters: basal oxygen consumption rate (OCR), basal extracellular acidification rate (ECAR), the maximal respiratory capacity of mitochondria and the spare respiratory capacity. There was, however, a trend toward increased mitochondrial spare respiratory capacity (**Figure 5C**) and maximal respiratory capacity (**Figure 5D**) in the convalescent MIS-C group (not significant). There was no difference in the observed oxidative burst capacity between the two groups. Similarly, no difference was observed in the metabolic profile of PMA and ionomycin activated PBMCs (**Figure S9**). Altogether, PBMCs or PMA/ionomycin stimulated PBMCs of convalescent MIS-C patients showed comparable metabolic phenotype and mitochondrial functions as the PBMCs from HC, and comparable oxidative burst capacity.

#### 3.6.2 Immunometabolism and mitochondrial function of monocytes and lymphocytes

Monocytes from COVID-19 patients have significantly impaired functionality, reduced spare respiratory capacity and impaired oxidative burst (19). We therefore characterized the immunometabolic profile of monocytes isolated from PBMCs. Altogether, the results did not show any major differences in the metabolism of monocytes of convalescent MIS-C patients compared to HC (**Figure S10**). We did, however, observe a weak trend of higher basal ECAR and ATP production from the oxidative phosphorylation (OxPhos) in the convalescent MIS-C group compared to the control group, but the differences were not significant (p > 0.05, Mann-Whitney test) (**Figures S10B, E**). We could observe an oxidative burst after injection with PMA and ionomycin in isolated monocytes (**Figure S10H**), however, there was no significant difference in the oxidative burst between the PA group and HC with a slight trend (not significant - ns) toward higher oxidative burst in the convalescent (14) MIS-C patients.

Measurements of lymphocytes isolated from PBMCs (**Figure S11**) showed a very low metabolic rate with low respiration (basal OCR) and low values of basal ECAR compared to isolated monocytes or PBMCs in accordance with higher metabolic demands of monocytes in relation to non-activated lymphocytes. Altogether, no significant differences were observed between the immunometabolism of lymphocytes from convalescent MIS-C patients and HC in any of the measured parameters, though we did observe a trend towards lower baseline ECAR and glycolytic ATP production in PA group (**Figure S11**). Expectedly, the basal levels of OCR and ECAR of isolated monocytes were much higher compared to levels obtained in isolated lymphocytes (**Figures S10A, B**, **S11A, B**).

#### 3.6.3 Immunometabolism and mitochondrial function of aCD3/aCD28 stimulated PBMCs – effects of MIS-C on T cell immunometabolism

**Figure 6:**
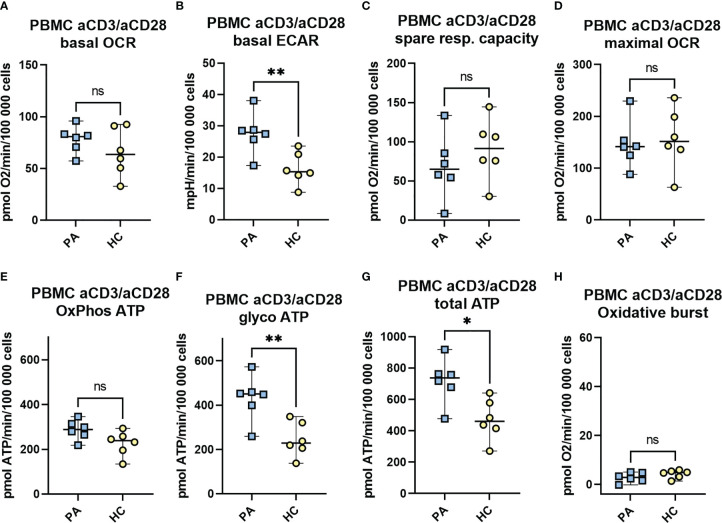
The metabolic profile and the mitochondrial function of aCD3/aCD28 activated PBMCs in convalescent phase. Isolated PBMCs of MIS-C patients in convalescent phase (PA, n = 6) and healthy controls (HC, n = 6) were seeded at a density of 10^6^ cells/mL in RPMI medium with 10% FBS and stimulated with CD3 and CD28 antibodies. Following 48h incubation, cells were seeded on a Seahorse plate at a density of 3 × (200 000 - 250 000) cells/well and OCR **(A)** and ECAR **(B)** were measured with Seahorse XFe24 analyzer under basal conditions, and in response to mitochondrial inhibitors (Mito Stress Assay): oligomycin (1.5 μM), FCCP (2 μM), and antimycin A plus rotenone (0.5 μM). The spare mitochondrial capacity **(C)** and maximal respiration - maximal OCR **(D)** were determined according to Mito Stress Assay, OxPhos ATP **(E)**, glycoATP **(F)** and total ATP **(G)** were calculated from OCR and ECAR measurements as defined in M&M. The oxidative burst **(H)** was measured after last injection with PMA/ionomycin (1 μg/ml). Data are presented as median with 95%CI and analyzed by Mann-Whitney test where *P < 0.05, **P < 0.01, ns, not significant. OCR, oxygen consumption rate, ECAR, extracellular acidification rate.

Next, we evaluated if the T lymphocytes of convalescent MIS-C patients after activation exhibit any differences in the metabolic profile compared to the healthy controls. We used activated PBMCs with anti-CD3 and anti-CD28 antibodies for 48 hours. The metabolic assay (Mito Stress Assay with the fourth injection of PMA/ionomycin) was performed 48 hours after the addition of aCD3/aCD28. Overall, the majority of the measured signal in aCD3/aCD28 activated PBMCs came from activated T lymphocytes. The measurements expectedly showed a higher metabolic rate compared to non-activated PBMCs with a markedly increased rate of OCR, ECAR and glycolytic ATP production (**Figure 6**).

Interestingly, both the basal level of ECAR and the rate of ATP production from glycolysis were significantly increased (2-fold increase) in convalescent MIS-C group (PA) compared to the healthy controls (**Figures 6B, F**). In addition, a trend toward increased rate of basal OCR and ATP produced by oxidative phosphorylation (OxPhos ATP) in PA (ns) (**Figure 6A, E**) and a weak trend toward decreased mitochondrial spare capacity (ns) were observed (**Figure 6C**). Finally, the total ATP production was significantly increased in the PA group compared to HC (**Figure 6G**).

### 3.7 Correlation analysis

In order to gain additional insight into possible associations between the clinical, immunological and metabolic parameters we present Spearman correlation coefficients (**Figure S12**) for the selected parameters. We observed positive correlation between TCRα/β in the acute phase and the number of the affected organ systems, while memory B lymphocytes showed negative correlation. DNTα/β correlated positively with peak BNP and troponin. In convalescent phase (PA) percentage of classical monocytes correlated with CRP while HLA-DR MFI of classical monocytes correlated negatively. Due to the limited number of patients (n=14) the observed correlations has to be interpreted with caution as it could be obtained by chance, especially for the metabolic parameters where n=6.

### 3.8 Characteristics of patients that required intensive care treatment and/or IL-1 antagonist treatment

One of the two patients who required care in an intensive care unit and was also treated with IL-1 receptor antagonist had the maximal values of peak troponin in patients group, very high BNP, high values of pDC, the lowest values of basal OCR and maximal OCR in PBMC and maximal values of OxPhosATP and GlycoATP in CD3/CD28 stimulated PBMC. The other patient that required intensive care had the lowest concentration of TCRγ/δ, the lowest CD21- B lymphocytes and CD21+ B lymphocytes and the lowest maximal OCR in CD3/CD28 stimulated PBMC.

## 4 Discussion

Several studies have shown marked but transient immune activation during MIS-C (15, 29–31). However, the specifics of immune functions in the acute phase and especially in the convalescent phase remain poorly understood. As the metabolic fitness of immune cells, especially T cells, has become one of the crucial markers of immune system (32), it is also important to determine the metabolic phenotypes of immune cells in the convalescent MIS-C to identify possible residual changes of hyperactivation and severe inflammation. To this end, we performed a longitudinal study with immunophenotyping of PBMCs of MIS-C patients in the acute and convalescent phases, In a subgroup of convalescent MIS-C patients we also performed metabolic profiling of PBMCs, monocytes, lymphocytes, and aCD3/aCD28 activated PBMCs.

Similar to other studies, we observed a decrease in major lymphocyte populations (B cells, CD4 + and CD8 + T cells, and NK cells) compared with the convalescent phase and healthy controls (**Figure 1**) (7, 29). Several studies have indicated that defective T cell responses are a key element of severe COVID-19 disease in adult patients (19, 20). In addition, we observed a decreased percentage and concentration of RTE (CD31 + CD45RA +) T cells, which could suggests a transient thymic insufficiency during the acute phase (**Figure 2C**), but this should be further confirmed by T-cell receptor circuits analysis (TRECS). Alternatively, reduced RTE concentrations could be explained by the shift of T helper cells towards a more activated or memory phenotype that occurs during inflammation with a loss of the CD31 + marker, although in our case we did not observe changes in the percentage of naive and activated or memory cells (**Figures 2A, B**) (33–35). Although the total concentration of CD3+ T cells did not fully recover to the level of healthy control subjects, the concentrations of major lymphocyte populations largely (T helper cells, NK cells, and RTE) or fully (B and cytotoxic T cells) returned to normal in the convalescent phase. These results suggest that lymphocyte populations are likely to recover in convalescent MIS-C patients, although recovery is not complete two to six months after diagnosis.

An important indication of the ongoing response to the strong immune activation and transient insufficiency in the acute phase of MIS-C is the increased proportion of double-negative T cells (DN T). These T cells have lost expression of CD4 and CD8 co-receptor but still express a TCR consisting of an α/β- or γ/δ-chain (**Figures 2E, F**), which enables them to recognize and further respond to pathogens (36). DN T cells have been characterized in various chronic inflammatory diseases and have been suggested to have pathogenic or regulatory functions (37). They are involved in the maintenance of both innate and adaptive responses and modulate the functions of macrophages, CD8+ T cells, and B cells (38). Voelkl et al. demonstrated that DN T exerts a strong suppressive effect on CD4+ and CD8+ cells (39). Based on current knowledge, DN T cells are probably generated in the periphery and differ from conventional regulatory T cells in that they inhibit the activation of early T cells independently of FoxP3 (40, 41).

Interestingly, we observed a significantly increased proportion of γ/δ DN T cells only in the convalescent phase of MIS-C in contrast to the acute phase (**Figure 2F**). They possess immunosuppressive capabilities and probably act as a negative regulator (36). It was shown that a high frequency of γ/δ DN T cells is associated with a lack of hepatitis B virus (HBV) control through suppression of CD8+ T cell responses to HBV antigens (36). Pro-inflammatory cytokines or TCR γ/δ activation may also increase the frequency of γ/δ DN T cells in peripheral blood (37). The increased level of γ/δ-DNTs could therefore represent a sustained adaptive response to the severe inflammation and activation of the immune system in acute MIS-C.

On the other hand, we observed an increased proportion of α/β DN T cells in both the acute and convalescent phases of MIS-C compared with healthy controls (**Figure 2E**). α/β DN T cells show a pro-inflammatory cytokine profile (40). The increased proportion of α/β DN T cells is therefore probably associated with the severe inflammation in the acute phase and, interestingly, is one of the immune changes that persist in the convalescent phase of MIS-C.

Another important parameter for the activity of the adaptive immune response is the percentage of activated T cells. In the acute phase of MIS-C, T cells were highly activated (HLA-DR positive), which is consistent with the observation by Vella et al. that CD8 + T cells were more activated in MIS-C patients compared with healthy controls (29). Conversely, the proportion of HLA-DR positive T cells normalized in the convalescent phase (**Figure 2D**). In addition, there were no differences in the metabolic profiles of lymphocytes isolated from PBMCs of patients in the convalescent MIS-C phase and healthy controls, with very similar values of glycolytic rate and oxygen consumption rates (**Figure S11**). Since T cell activation and effector functions such as IFN-γ secretion are associated with increased respiration (42) and glycolysis (43), respectively, this is further evidence of largely normalized T cell activation in the convalescent phase of MIS-C.

To better understand possible changes in T cells or to observe markers of T cell exhaustion, the metabolic profile of activated T lymphocytes was measured in the convalescent MIS-C phase. The metabolic profile of CD3/CD28 activated PBMC was measured after 48 hours, with activated T cells providing most of the measured signal. Interestingly, activated T cells from PBMCs of convalescent MIS-C patients showed a significantly increased extracellular acidification rate (P < 0.01) (**Figure 6B**) and a trend toward an increased OCR rate (ns) in the convalescent phase compared with healthy controls (**Figure 6A**). This was also reflected in increased ATP production by glycolysis (P < 0.01) (**Figure 6F**) in convalescent MIS-C group compared with HC. These results suggest that T lymphocytes in the convalescent MIS-C phase have some residual changes that lead to higher metabolic activity when activated. One of the reasons could be that after long-term activation of T lymphocytes, the expression of CD3 and co-receptors CD4, CD8 are reduced (**Figure S6**), but the level of transcription factor NF-kB, phosphatases, and cytokines in lymphocytes is still increased, so they respond strongly to CD28 and CD3 stimulation with nonlateral signals. Immunometabolic studies have shown that the metabolism of T cells is closely related to their activation (32). Activated T cells have increased glycolytic rate and increased OxPhos, which provides intermediates and ATP for rapid proliferation and their normal functionality in the form of cytokine secretion (43). Thus, the increased rate of glycolytic ATP production and a trend toward increased OxPhos in PBMCs of convalescent MIS-C patients indicate that these cells respond more intensely to the stimulus compared with PBMCs in the healthy controls. In relation to the immunophenotyping results, it suggests that this may be directly due to changes in the T cells populations themselves or due to the observed differences in pDC cells in the convalescent phase. Alternatively, the greater upregulation of energy metabolism after activation, as well as some of the differences observed between the convalescent group and HC group, may be an intrinsic feature of T cells in children susceptible to developing MIS-C after SARS-CoV-2 infection, possibly in association with some genetic predispositions that contribute to the development of MIS-C (11, 12).

Other studies have shown dysregulations in peripheral B cell differentiation, with an increased proportion of naive B cells and a decrease in memory B cells in patients with acute MIS-C compared with healthy controls (7). We also observed a marked reduction in proportion of memory B cells and memory-switched B cells (CD27+CD19+CD21+IgM-IgD-) in acute MIS-C versus healthy control group, but the proportions mostly returned to normal in the convalescent phase. In acute MIS-C, we observed a decreased proportion of functional B cells expressing the complement receptor CR2 (CD21). The reduction of proportion of CD21+B cells results in an impaired humoral response to T dependent antigens, characterized by a reduction in B cell follicle retention and germinal center survival (44). This indicates a moderate maturation arrest of B cells in the acute phase (**Figure 3**). In the convalescent phase, the proportion of B cell subpopulations usually returns to the normal range.

Interestingly, many studies reported an increased percentage of plasmablasts in the acute MIS-C (7, 45), whereas we observed a statistically significant reduction in the convalescent phase. The reduced percentage is probably transient and could be due to increased apoptosis during the period when the immune response returns to normal (46). On the other hand, MIS-C often presents as inflammation of the mucosal tissue, vascular wall, and other tissues with a marked presence of B-cell infiltrates. Therefore, a significantly reduced proportion of activated B cells and memory-switched B cells in acute MIS-C may reflect infiltration to the site of inflammation (47).

In the acute phase of MIS-C, we also observed a significantly decreased concentration of total monocytes compared with the concentration in the convalescent phase (**Figure S10I**). Classical monocytes play an important role in inflammation and its resolution. They have the ability to differentiate into monocyte-derived macrophages and DCs (28). We found a significantly lower proportion of classical monocytes in the acute phase of MIS-C (**Figure 4D**), which could be due to chemoattraction and migration from the blood to the inflamed tissue (4).

It has been previously reported that HLA-DR is downregulated in monocytes from MIS-C patients compared with healthy controls (14, 26). We also observed similar tendency for downregulation of HLA-DR in classic monocytes during acute phase, which normalized in the convalescent phase. This is consistent with observed downregulation of HLA-DR in cases of systemic inflammation, representing a type of immunoparalysis (48).

The concentration of total monocytes returned to normal values in the convalescent phase. Similarly, the metabolic profile of monocytes in the convalescent phase was very similar to HC (**Figure S10**). The only potential metabolic indication of residual changes in monocytes was a weak trend toward higher oxidative metabolism (OxPhos ATP production) (**Figure S10E**). Another key parameter reflecting the functional capabilities of monocytes is the oxidative burst (19). We obtained a similar oxidative burst in monocytes of convalescent MIS-C patients (**Figure S10H**) compared to HC, indicating a return to normal monocyte functionality (49) in contrast to monocytes from acute COVID -19 patients (19), in which a defective respiratory burst was observed. Overall, both immunophenotyping and metabolic profiling results suggest that monocyte populations and their functional capacity return to their normal state two to six months after the acute MIS-C phase, with some evidence of slightly increased oxidative metabolic activity remaining.

As dendritic cells constitute only a very small proportion of PBMCs, we could not measure metabolic profile on isolated DCs. However, we performed immunophenotyping to distinguish different subsets of DCs: conventional type 1 DCs (cDC1s), conventional type 2 DCs (cDC2s), and plasmacytoid DCs (pDCs). pDCs are important for early control of viral infections, and deficiency of pDCs not only leads to uncontrolled viral replication and spread to different organs but also affects the severity of viral diseases (30). Overall, the percentage of pDCs was significantly reduced in both the acute and convalescent phases compared with HC, with a tendency to normalize in the convalescent phase (**Figure 4**). The frequency of pDCs was greatly reduced in the acute MIS-C compared with HC (0.5% of all PBMC which were HLA-DR+ CD14- cells versus 3.4% in HC, P < 0.0001, **Figure 4C**). Our results are in agreement with other studies that also showed a lower number of pDCs in the acute phase of MIS-C (4, 26) and in adult patients with severe COVID -19 (27). This could be due to mass activation of pDCs leading to apoptosis or recruitment to the inflammation site (4). Interestingly, we also observed a decreased proportion of pDCs in the convalescent phase, with a smaller but still significant difference compared with HC (1.4% vs. 3.4%, P = 0.047), which has not been described before. A similar trend was observed in adult patients with SARS-CoV-2 infection in the acute phase and 7 months after infection. Perez-Gomez et al. have showed that the alterations in DC subsets were associated with altered homing and activation patterns implicating ongoing inflammation (50). Importantly, pDCs express Toll-like receptor-7 (TLR -7), a receptor for single-stranded RNA, important for viral recognition and innate immune response. In addition, pDCs are the major source of type I IFNs and it was shown that pDCs play a crucial role in the pathogenesis of coronavirus infection (51). Additionally, study of respiratory syncytial virus showed that pDCs not only help minimize the immunopathologic damage associated with viral infection but also facilitate the establishment of antiviral T cell responses in the lung (52). It should be noted that neither macrophages, cDCs, fibroblasts, nor lung epithelial cells were able to elicit a significant IFN type I response to SARS-CoV-2 (24). The lack of a significant type I IFN response in SARS-CoV2-infected patients may be due to inhibition and/or modulation of the pDC response by various viral nonstructural proteins (4, 51).

Furthermore, we observed an increased proportion of cDC1s in the acute phase that normalized in the convalescent phase. Similar as in (26), we observed that type 1 dendritic cells (cDC1s) were upregulated in acute MIS-C patients, whereas type 2 dendritic cells (cDC2s) appeared to be reduced. cDC1s have the ability to initiate *de novo* T-cell responses after migrating to draining lymph nodes, as well as to attract T cells, secrete cytokines, and present viral antigens in the inflamed microenvironment, enhancing local cytotoxic T-cell function. cDC1s recognize viral antigens and trigger type 1 immune responses, including induction of T helper cell 1, ILC1, and NK cells. In addition, cDC1 efficiently present extracellular antigens to CD8+ T cells and secrete IL-12, making them important for cytotoxic responses to viral infections (53). On the other hand, cDC2 are more specialized in polarizing CD4+ helper T cells and supporting B cells (54). In our study, both cDC populations largely returned to normal levels in convalescent MIS-C patients, indicating normalization of the imbalance observed in the acute phase.

Overall, most APC populations, including cDC and monocyte subsets, returned to normal levels in the convalescent phase of MIS-C, accompanied by a predominantly normal monocyte metabolic profile and oxidative burst function. However, recovery of pDC populations was incomplete even during the convalescent phase of the disease, suggesting that a suboptimal type I IFN response to viral infection persists. We should stress that one of the limitation of our study is small number of MIS-C patients and healthy controls, therefore further studies are needed.

## 5 Conclusions

The observed changes in cDC1 and cDC2 subpopulations show a unique pattern that may suggest that antigen cross-presentation is part of the immune dysregulation of MIS-C even months after the acute phase of the disease. In further studies, it would be interesting to investigate whether a severely reduced proportion of pDCs has diagnostic predictive value that children with SARS-CoV2 infection will develop MIS -C. Other characteristic features of the acute phase of MIS-C were lymphopenia, delayed T-cell differentiation, and the occurrence of strong T cell activation with the consequent formation of residual α/β DN T and γ/δ DN T cells. This was accompanied by a disruption of B lymphocyte differentiation manifested as a decreased proportion of memory, memory-switched, and non-switched B cells and a decreased proportion of B cells expressing the complement receptor CR2 (CD21).

Remarkably, the impaired regulation of the immune system was evident in the convalescent phase of the disease even months after the acute phase, and was manifested as an increased proportion of γ/δ DN T cells, a decreased proportion of plasmablasts, and a decreased proportion of pDCs in the blood of convalescent children. These results were further supported by the observed increased rate of glycolysis of PBMCs stimulated with aCD3/aCD28 antibody in the convalescent phase, indicating increased upregulation of energy metabolism after stimulation. Most of the other observed changes in the immune cell populations and immunometabolic parameters in the acute phase were largely or completely normalized in the convalescent phase two to six months after the onset of acute disease.

Our data support the model that acute and life-threatening postinfectious inflammatory episodes of MIS-C cause long-term immune changes in the fraction of children and adolescents that are observed even several months later in the convalescent phase. Although our findings in MIS-C require a larger number of patients for further testing, our observations aid to understanding of the mechanism of MIS-C and may help to improve immunological follow–up of patients with MIS-C.

## Data availability statement

The original contributions presented in the study are included in the article/**Supplementary Material**. Further inquiries can be directed to the corresponding authors.

## Ethics statement

The studies involving human participants were reviewed and approved by the Slovenian National Medical Ethic Committee (approval number 0120-211/2020/7). The patients/participants provided their written informed consent to participate in this study. Written informed consent to participate in this study was provided by the participants’ legal guardian/next of kin.

## Author contributions

ANK, MP, AI and TA designed the study. JR, LJ, ANK and MP performed the experiments, data analysis and interpretation. MB, TV, NE, MZ and TA included patients in the study and obtained clinical information. ANK and MP wrote the original draft. All authors contributed to the article and approved the submitted version.
