# Supplementary material for: Alterations in immunophenotype and metabolic profile of mononuclear cells during follow up in children with multisystem inflammatory syndrome (MIS-C)
Source: Front Immunol. 2023 Apr 20;14:1157702. doi: 10.3389/fimmu.2023.1157702 (PMC10157053; doi:10.3389/fimmu.2023.1157702)
Supplement: Supplementary file 1 [file DataSheet_1.pdf]

Supplementary material

TABLE S1: KEY RESOURCES:

ANTIBODIES	CLONE	VOL./TEST (µL)	SOURCE	IDENTIFER
**Anti-Human CD45 V500**	HI30	5	BD Bioscience	560777
**Anti-Human CD3 PE Cy7**	UCHT1	5	eBioscience	25-0038-42
**Anti-Human CD4 V450**	SK3	5	BD Bioscience	651849
**Anti-Human CD8 APC Cy7**	SK1	5	BD Bioscience	348813
**Anti-Human CD19 PE-Cy7**	SJ25C1	5	BD Pharmingen	557835
**Anti-Human CD21 APC**	B-ly4	10	BD Pharmingen	559867
**Anti-Human CD27 PerCP Cy5.5**	M-T271	5	BD Pharmingen	560612
**Anti-Human CD38 V450**	HB-7	5	BD Bioscience	646851
**Anti-Human IgD PE**	IA6-2	10	BD Pharmingen	555779
**Anti-Human IgM**	G20-127	15	BD Pharmingen	555782
**Anti-Human TCRαβ FITC**	T10B9	10	BD Bioscience	555547
**Anti-Human TCRγδ PE**	11F2	10	BD Bioscience	333141
**Anti-Human CD14 PerCP Cy5.5**	M5E2	10	BD Pharmingen	550787
**CD16 PE**	3G8	10	BD Pharmingen	555407
**Anti-Human HLA-DR PerCP Cy5.5**	L245	10	BD Bioscience	339216
**Anti-Human HLA-DR APC Cy7**	L243	5	BD Bioscience	335831
**Anti-Human CD31 APC**	WM-59	5	eBioscience	17-0319-42
**Anti-Human CD1c BV510**	F10721A3	5	BD Bioscience	742747
**Anti-Human CD45RA BV510**	HI100	5	BD Bioscience	563031
**Anti-Human CD11c APC**	MJ4-27G12	10	Macsmilteny Biotec	130-113-576
**Anti-Human CD303 FITC**	AC144	10	Macsmilteny Biotec	130-133-192
**Anti-Human CD123 PE-Cy7**	7G3	5	BD Pharmingen	560826
**Anti-Human CD141 VioBlue**	ADS-14H112	10	Macsmilteny Biotec	130-113-320
**BD Multitest 6 col. TBNK Trucount Tubes**		20	BD Bioscience	340504

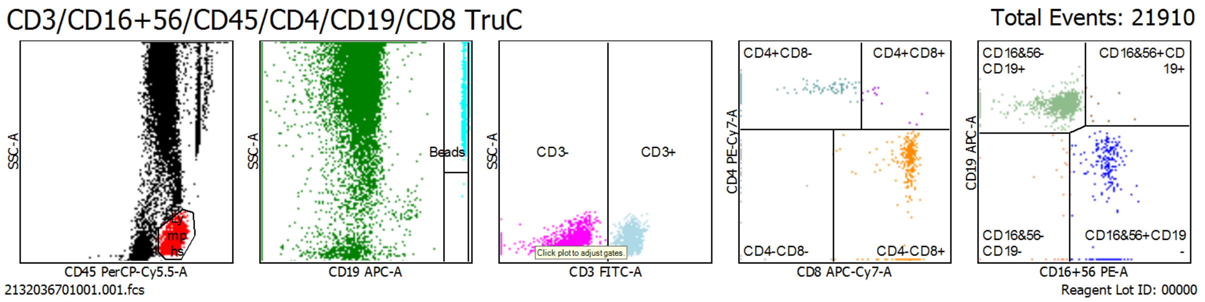

Parameter	Percent	Value/AbsCnt
Lymph Events		2450
Bead Events		3024
CD3+	51.18	406.80
CD3+CD8+	16.73	133.01
CD3+CD4+	31.67	251.74
CD3+CD4+CD8+	0.53	4.22
CD16+CD56+	11.63	92.46
CD19+	35.71	283.85
CD45+		794.79
4/8 Ratio		1.89

Figure S1: A representative BD FACSCanto laboratory report showing with data from major lymphocyte populations collected using BD Trucount tubes. A blood sample stained with BD Multitest™ 6-color TBNK reagent in a BD Trucount™ tube was collected using a BD FACSCanto II flow cytometer. Analysis was performed automatically using BD FACSCanto v 3.0 software. On the CD45 vs. SSC dot plot, lymphocyte populations were identified as bright, compact clusters with low SSC. The percentage of lymphocyte subsets and absolute counts were automatically calculated and beads were identified on the CD19 vs SSC dot plot. In the next step, T cells were automatically separated from other CD3-negative lymphocytes. CD3-positive T cells were then further analyzed based on CD4 and CD8 expression (helper T cells, cytotoxic T cells), and CD3-negative cells were identified as B cells or NK cells based on CD19 or CD56+CD16 expression. The table shows the proportions and concentrations of the major lymphocyte populations in total lymphocytes (CD45+ cells, low SSC). A representative example of blood analysis from a MIS-C patient in the acute phase is presented.

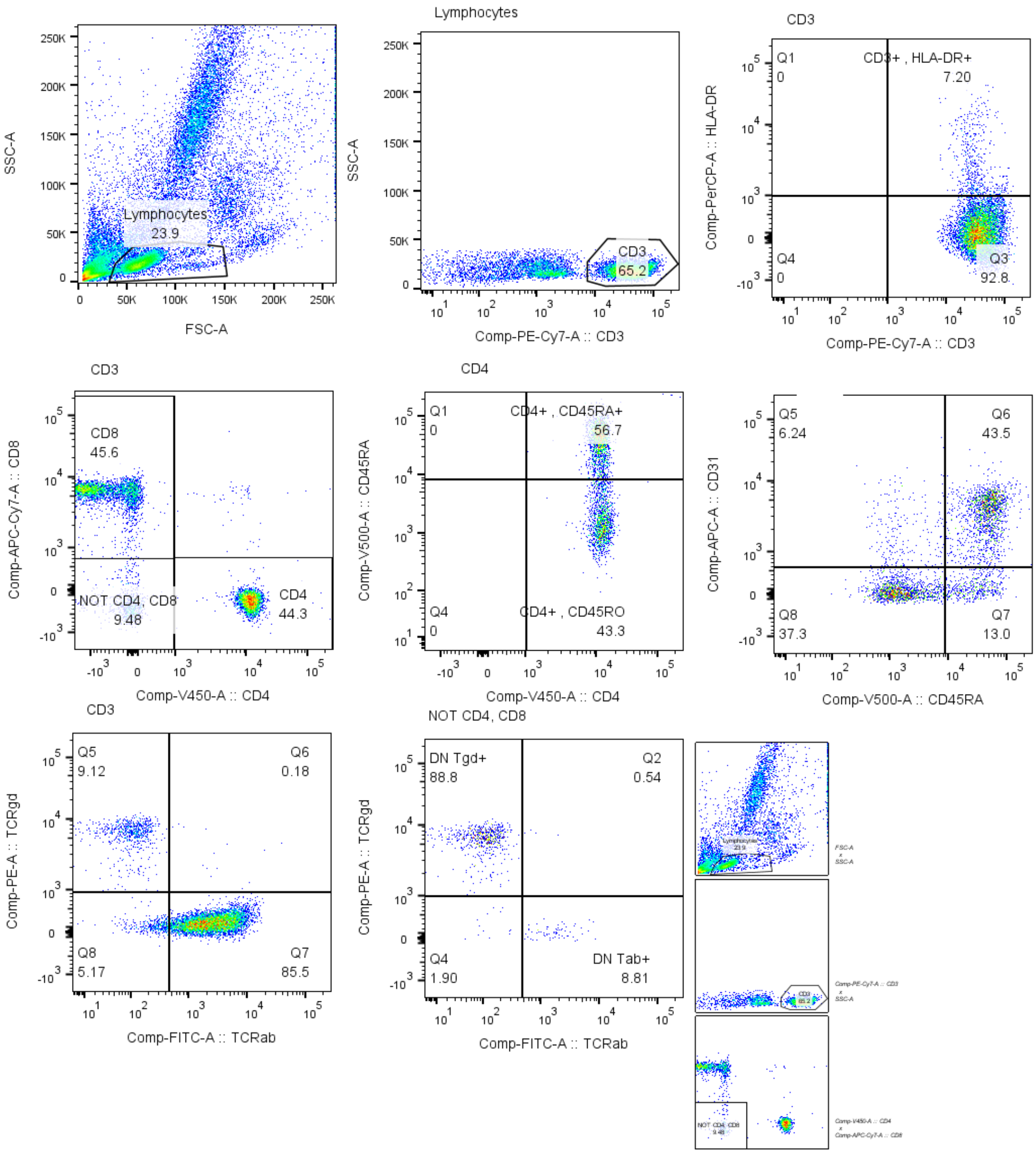

Figure S2. Gating strategy for the identification of T cells subpopulations. Among lymphocytes identified by size and complexity (FSC vs SSC), T cells were identified by the expression of CD3 in a pseudocolor dot plot CD3 vs SSC. Among T cells, activated T cells (HLA-DR +), TCR-α/β and TCR-γ/δ T cells were determined. Among helper T cells, naive and memory T cells and RTE were determined. DN T-α/βand DN T -γ/δT cells were determined within T cells not expressing CD4 and CD8. Statistical analysis of DN T cells considered the frequency of the grandparent population (CD3+ cells). A representative example of blood analysis from a patient in the convalescent phase of the disease is presented.

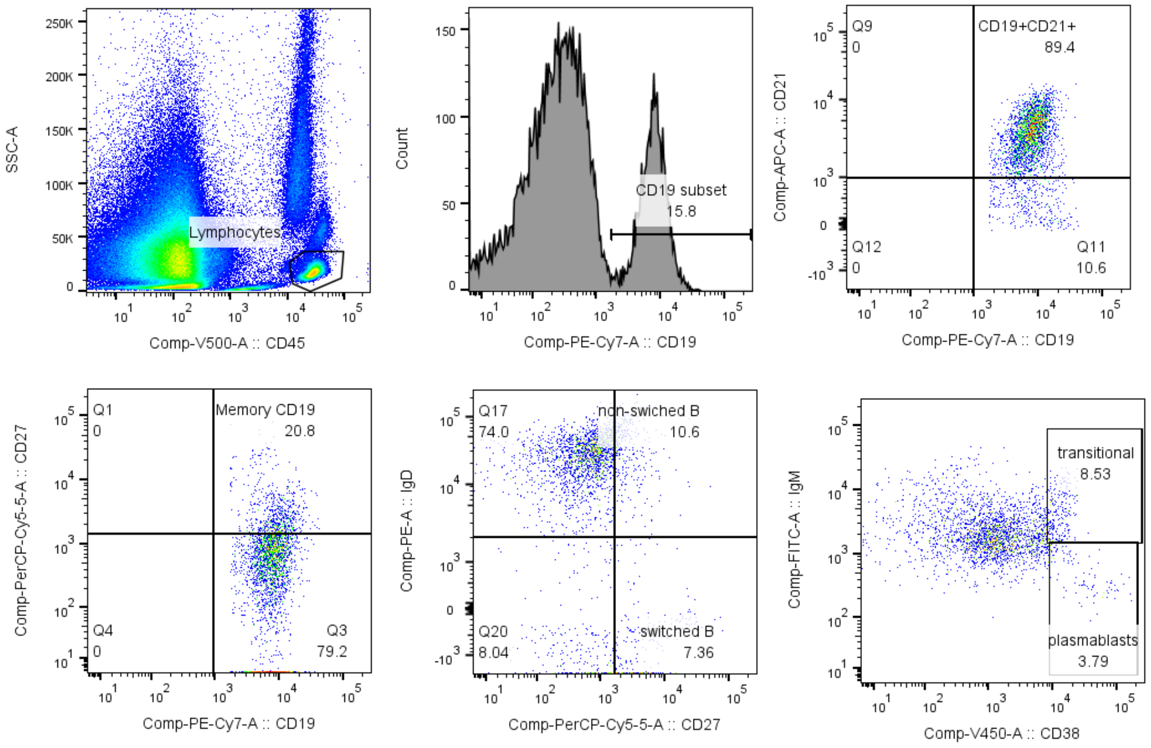

Figure S3. Gating strategy for identification of B-cell subpopulations. On the CD45 vs SSC dot plot, lymphocyte populations were identified as a bright, compact cluster with low SSC. Within lymphocytes, B cells were identified by expression of CD19 on a histogram. Among B cells, CD21-positive B cells, memory B cells (CD27+), memory-switched B cells (CD27+, IgM-), and non-switched B cells (CD27+IgM+) were analyzed. Transitional B cells were identified as CD38-bright IgM-positive cells and plasmablasts as CD38-bright IgM dim cells. A representative example of blood analysis from a MIS-C patient in the convalescent phase is presented.

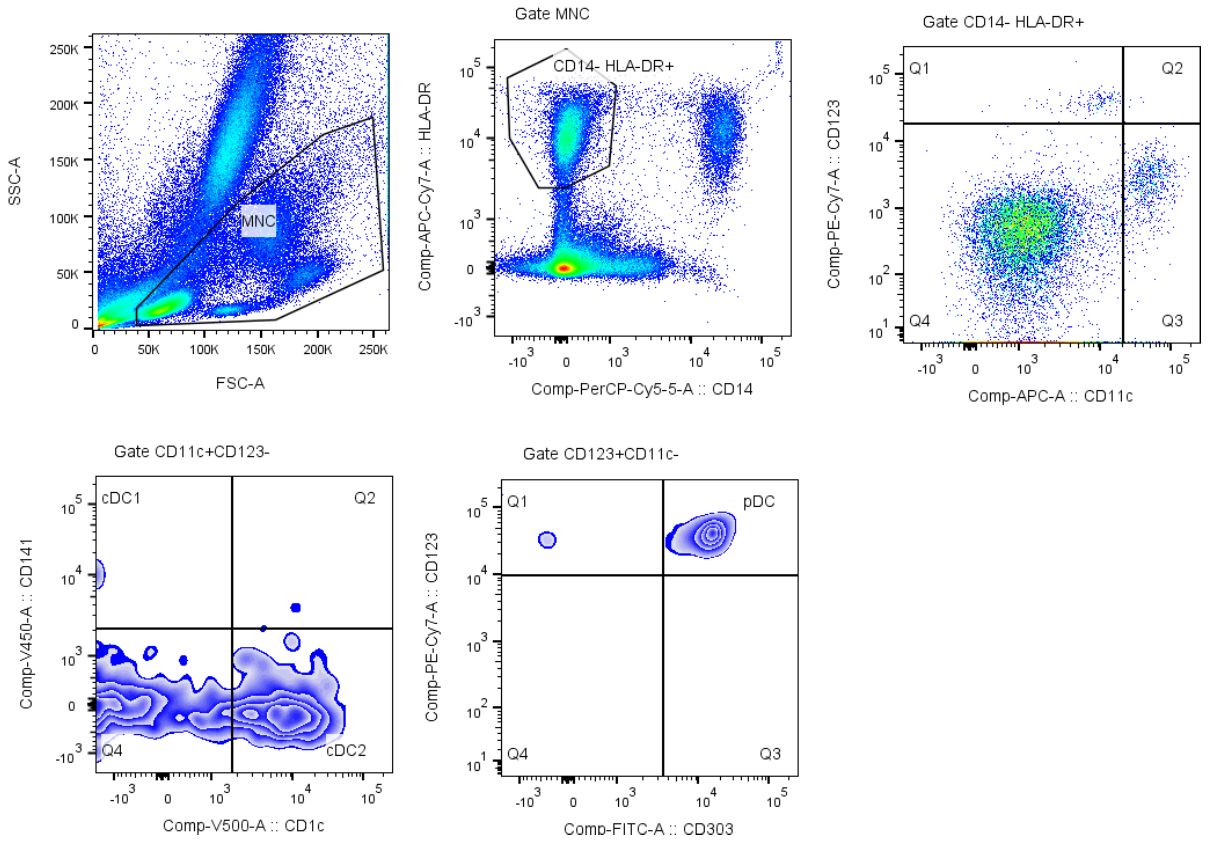

Figure S4: Identification of DC subpopulations. Pseudocolor dot plots showing the gating strategy used to identify the DC subpopulations. Mononuclear cells (MNCs) were selected based on their size (FSC-A) and complexity (SSC-A). DCs subpopulations were identified as follows: CD11c-positive CD123-negative cells within CD14- HLA-DR + were identified as cDCs, CD123 and CD303 positive cells within CD11c-CD123+ were identified as pDCs. Within cDCs: cDC2 (CD1c+) and cDC1 (CD141+).

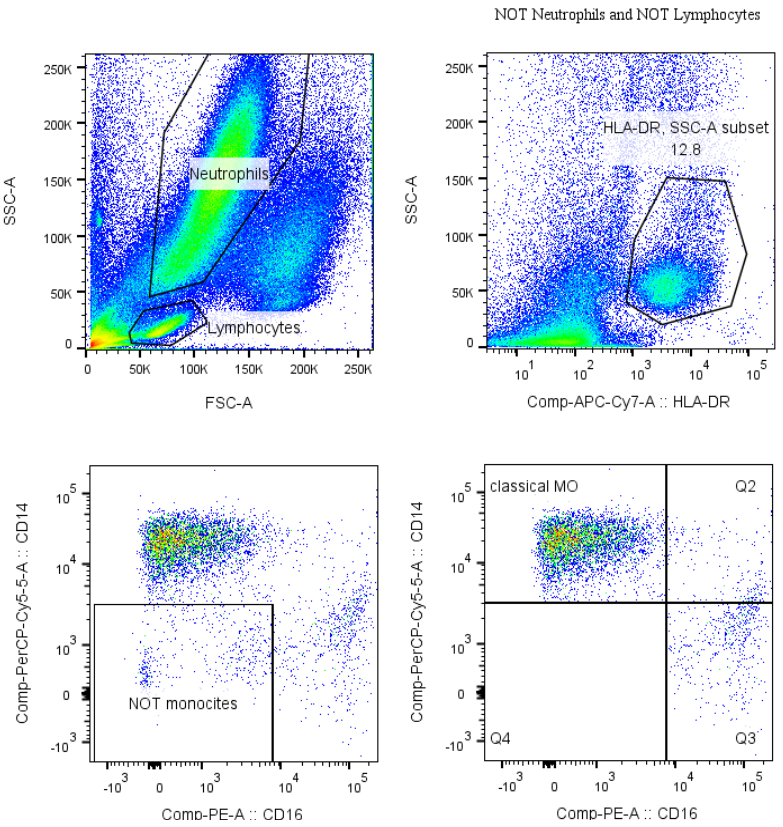

Figure S5: Identification of classical monocytes. Gating strategy to identify classical monocytes with successive exclusion of neutrophils and lymphocytes in conventional bivariate scatter plots of side scatter vs. forward scatter. The remaining population was selected as HLA-DR positive cells discriminated in a CD14 vs. CD16 pseudocolor dot plot to identify classic monocytes as CD14 positive and CD16 negative cells. Statistical analysis of classical monocytes considered the frequency of the grandparent population (HLA-DR+ cells).

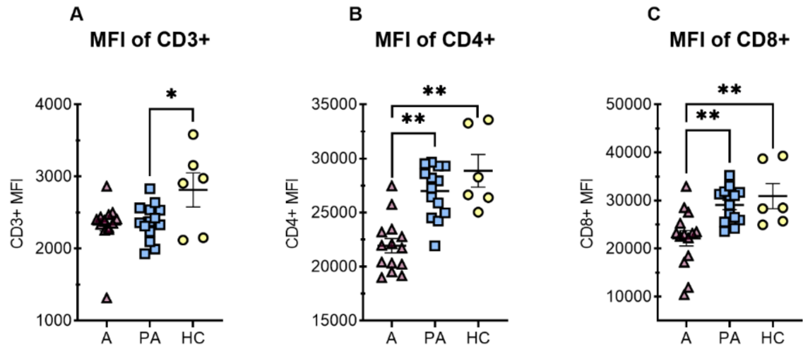

Figure S6: Median fluorescence intensities (MFIs) of total T cells, helper T cells and cytotoxic T cells. A-C, CD3+ T cells median fluorescence intensity (MFI). **(A)** CD3+ T cells MFI, **(B)** CD4+ T cells MFI and **(C)** CD8+ T cells MFI. Individual value plots show a dot for the actual value of each observation in a group. Pink triangles represent patients with acute MIS-C (A, n = 14 samples)), blue squares represent patients with convalescent MIS-C (PA, n = 14) and yellow dots represent healthy controls (HC, n = 6). Data are presented as means ± SEM; the Shapiro-Wilk normality test was performed. Significance testing between patients

by phase of illness (A- acute, PA – convalescent) was performed using paired t-test for normal distribution or Wilcoxon test for non-normal distribution. For comparison between patient groups and healthy controls (HC), *P ≤ 0.05 and **P ≤ 0.01 were determined by using un-paired t-test, if the distribution was normal and Mann-Whitney test for non-normal distribution.

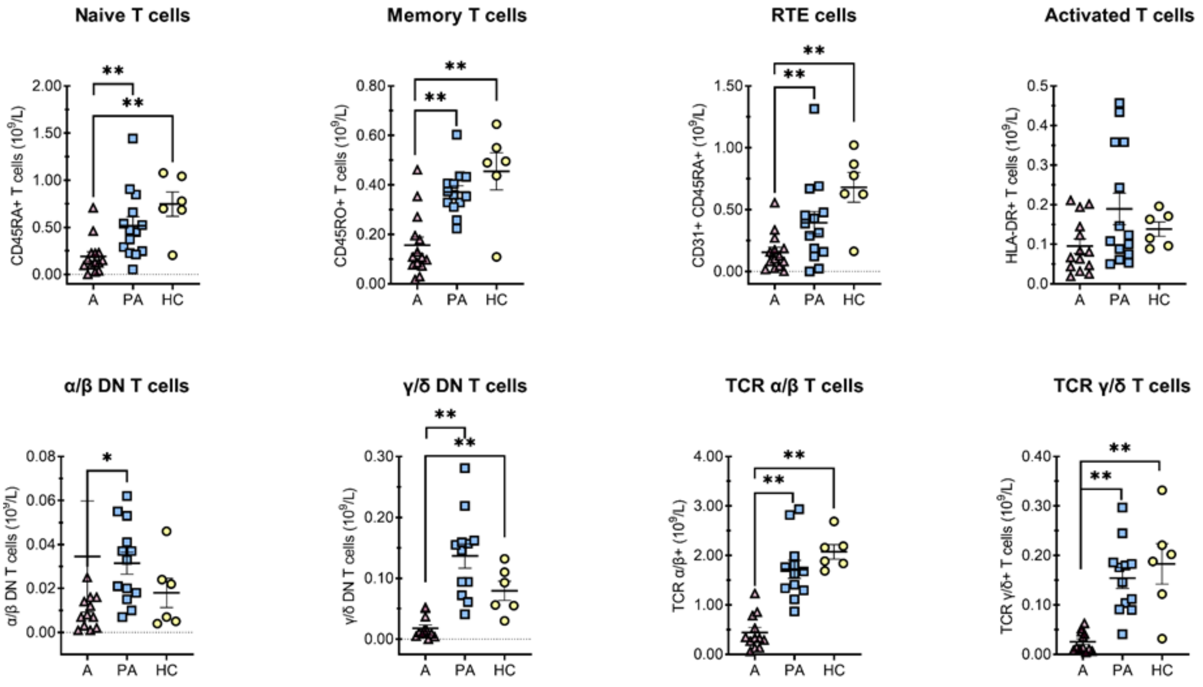

Figure S7: T cells differentiation in acute and convalescent phases of MIS-C. A-N, **(A)** Concentrations of T cells subpopulations: naive CD4+ T cells, memory CD4+ T cells, RTE - recent thymic emigrants, activated HLA-DR+ T cells, DN T α/β, DN T γ/δ, α/β T cells, γ/δ T cells× 10^9 per liter of blood. Individual value plots show a point for the actual value of each observation in a group. Pink triangles represent patients with acute MIS -C (A, n = 12-14), blue squares represent patients in convalescent phase of MIS -C (PA, n = 13-14), and yellow dots represent healthy controls (HC, n = 6). Data are presented as means ± SEM. The Shapiro-Wilk normality test was performed. Significance tests between patients by phase of illness (A – acute, PA - convalescent) were performed using the paired-samples t-test for normal distribution or the Wilcoxon test otherwise. For comparison between patient groups and HC, *P ≤ 0.05 and **P ≤ 0.01 were determined with an unpaired t-test if the distribution was normal and with the Mann-Whitney test otherwise.

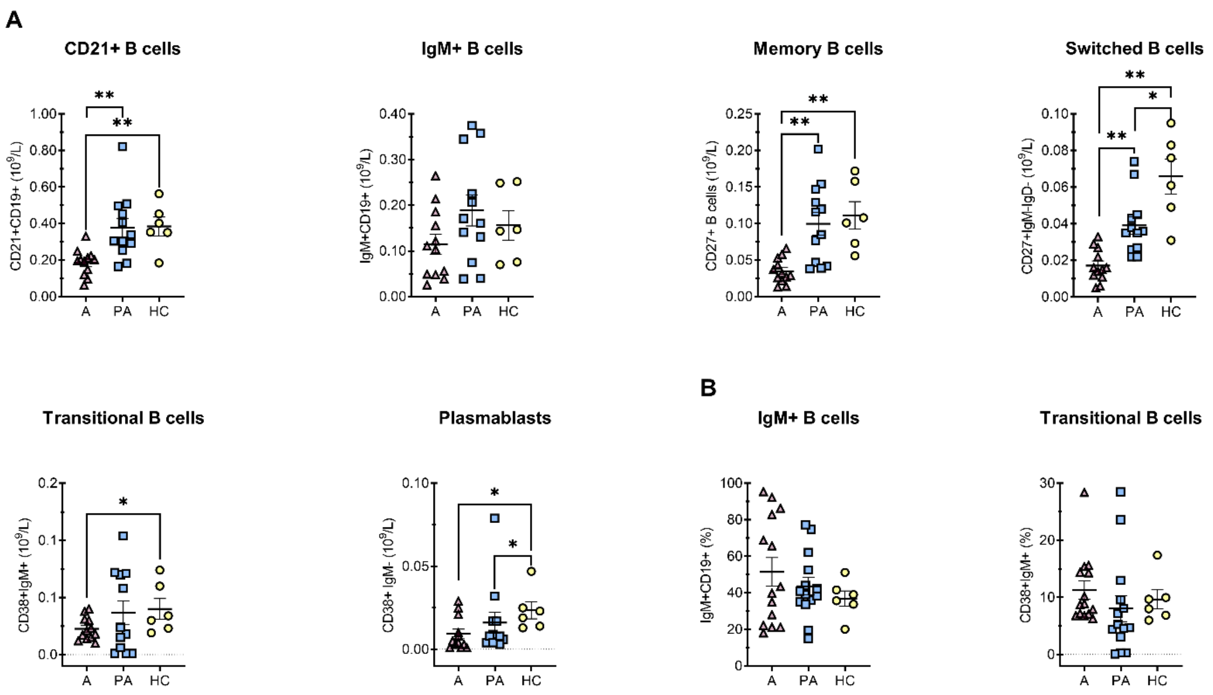

Figure S8: B cells differentiation in acute and convalescent phases of MIS-C compared to age-matched healthy controls. **(A)** Concentrations of B cells sub-populations: immunocompetent mature CD21+ B cells, IgM+ B cells, memory CD27+ IgM B cells, memory-switched B cells (Switched B cells), transitional B cells and plasmacytoid B cells (Plasmablasts) × 10^9 per liter of blood. **(B)** Percentages of B cells sub-populations: IgM+ B cells and transitional B cells. Individual value plots show a point for the actual value of each observation in a group. Pink triangles represent patients with acute MIS-C (A, n = 12-14), blue squares represent patients in convalescent phase of MIS-C (PA, n = 13-14), and yellow dots represent healthy controls (HC , n = 6). Data are presented as means ± SEM; the Shapiro-Wilk normality test was performed. Significance tests between patients by phase of illness (A – acute, PA - convalescent) were performed using the paired-samples t-test for normal distribution or the Wilcoxon test otherwise. For comparison between patient groups and HC, $^*P \leq 0.05$ and $^{**}P \leq 0.01$ were determined with an unpaired t-test if the distribution was normal and with the Mann-Whitney test otherwise.

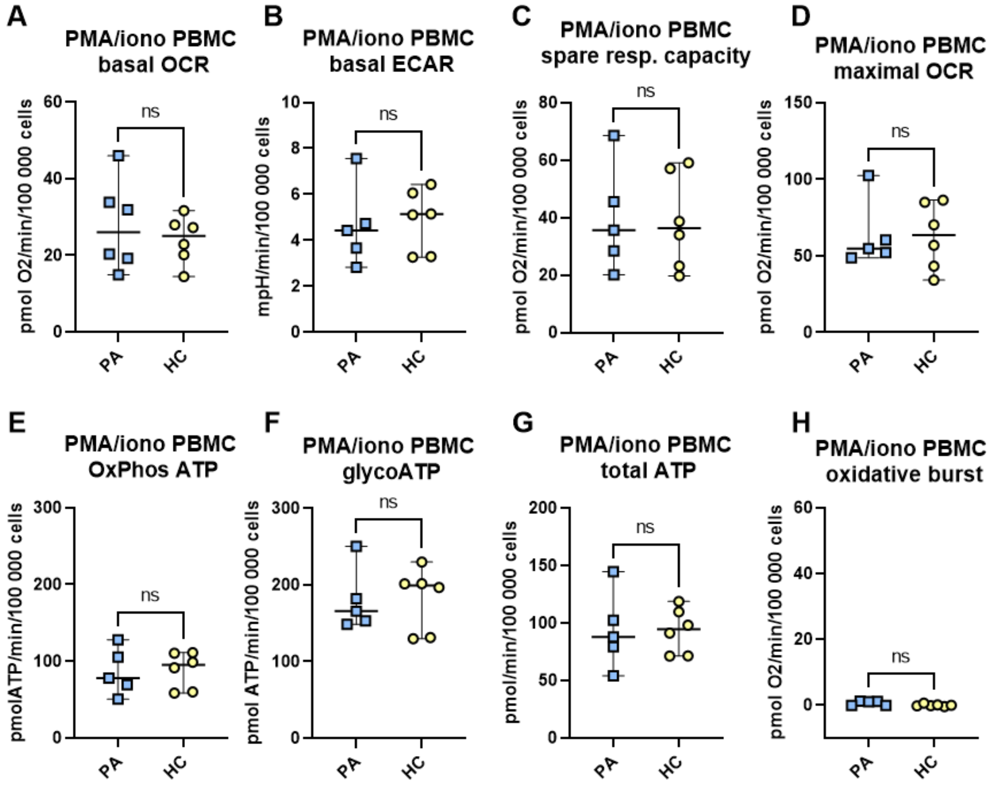

Figure S9: The metabolic profile and mitochondrial function of PMA/ionomycin activated PBMCs from patients with MIS-C in convalescent phase. The metabolic profile of PBMCs freshly isolated from peripheral blood of post-acute MIS-C patients (PA) and healthy donors (HC), treated for 4h with PMA/ionomycin. Following 4h treatment, OCR and ECAR were measured in real time, under basal condition, and in response to mitochondria inhibitors (Mito Stress Assay): oligomycin (1.5 μM), FCCP (2 μM), and antimycin A plus rotenone (0.5 μM). Oxidative burst was measured after last injection with PMA/ionomycin (PMA/Iono, 1 μg/ml), the maximal respiration (Maximal OCR) and spare mitochondrial capacity were determined according to the Mito Stress Assay. Data are presented as median with 95% CI and analyzed by Mann-Whitney test where *P < 0.05, **P < 0.01. OCR, oxygen consumption rate, ECAR, extracellular acidification rate.

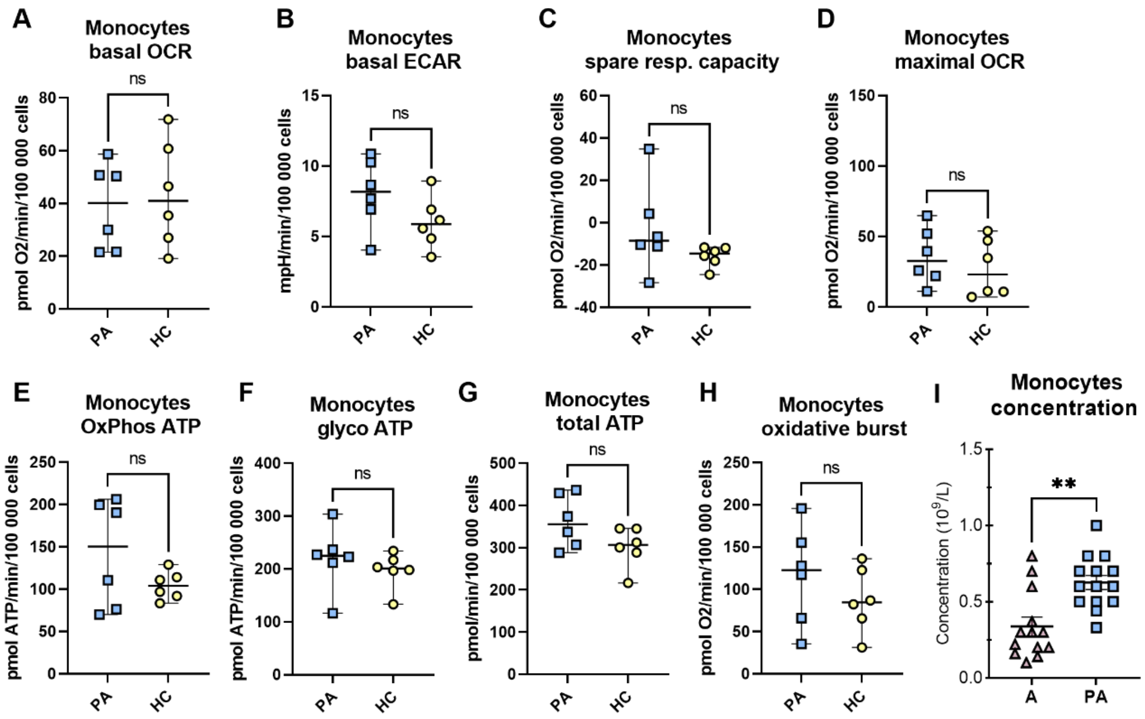

Figure S10: The metabolic profile in monocytes isolated from PBMCs of MIS-C patients in convalescent phase. (A-H) Monocytes were separated using magnetic beads from the isolated PBMCs obtained from MIS-C patients (PA, n = 6) in the convalescent phase and from the healthy controls (HC, n = 6). Separated monocytes were seeded in Seahorse plates. Following 4h incubation, OCR and ECAR were measured in real time, under the basal conditions and in response to the mitochondrial inhibitors (Mito Stress Assay): oligomycin (2 µM), FCCP (0.5 µM), and antimycin-A plus rotenone (0.5 µM). Oxidative burst was measured after last injection with PMA/ionomycin (1 µg/ml). Data are presented as median with 95%CI and analyzed by Mann-Whitney test where *P < 0.05, **P < 0.01. OCR, oxygen consumption rate, ECAR, extracellular acidification rate**. I** Concentration of total monocytes in patients with MIS-C in the acute (A) compared with post-acute phase (PA) $\times 10^9$ per liter of blood**.** Triangles - patients with acute MIS -C (A, n = 14) and squares represent convalescent atients with post-acute MIS-C (PA, n = 14). Data are presented as means ± SEM and were tested for normal distribution using the Shapiro-Wilk normality test. *P ≤ 0.05 was determined with Wilcoxon test.

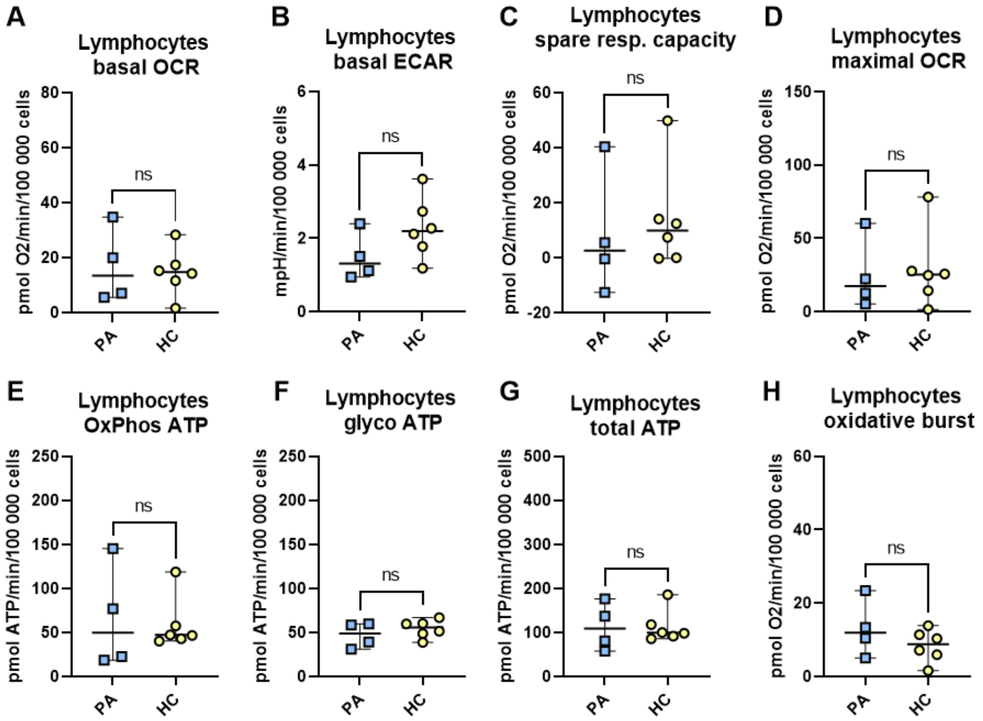

Figure S11: The metabolic profile and mitochondrial function in lymphocytes from PBMCs of patients with MIS-C in convalescent phase. Lymphocytes were obtained by magnetic separation of monocytes from freshly isolated PBMCs from MIS-C patients in post-acute phase (PA) and from healthy donors (HC). The remaining cells, mostly lymphocytes, were seeded on a Seahorse plate and following 4h incubation, Mito Stress Assay was performed. OCR and ECAR were measured in real time, under the basal condition, and in response to the mitochondrial inhibitors (Mito Stress Assay): oligomycin (1.5 µM), FCCP (2 µM), and antimycin A plus rotenone (Rot/AA, 0.5 µM). The oxidative burst was measured after the last injection with PMA/ionomycin (PMA/Iono, 1 µg/ml), the maximal respiration (Maximal OCR) and the spare mitochondrial capacity were determined according to the Mito Stress Assay. Data are presented as median with 95% CI and analyzed by Mann-Whitney test where $*P < 0.05$, $**P < 0.01$. OCR, oxygen consumption rate, ECAR, extracellular acidification rate.

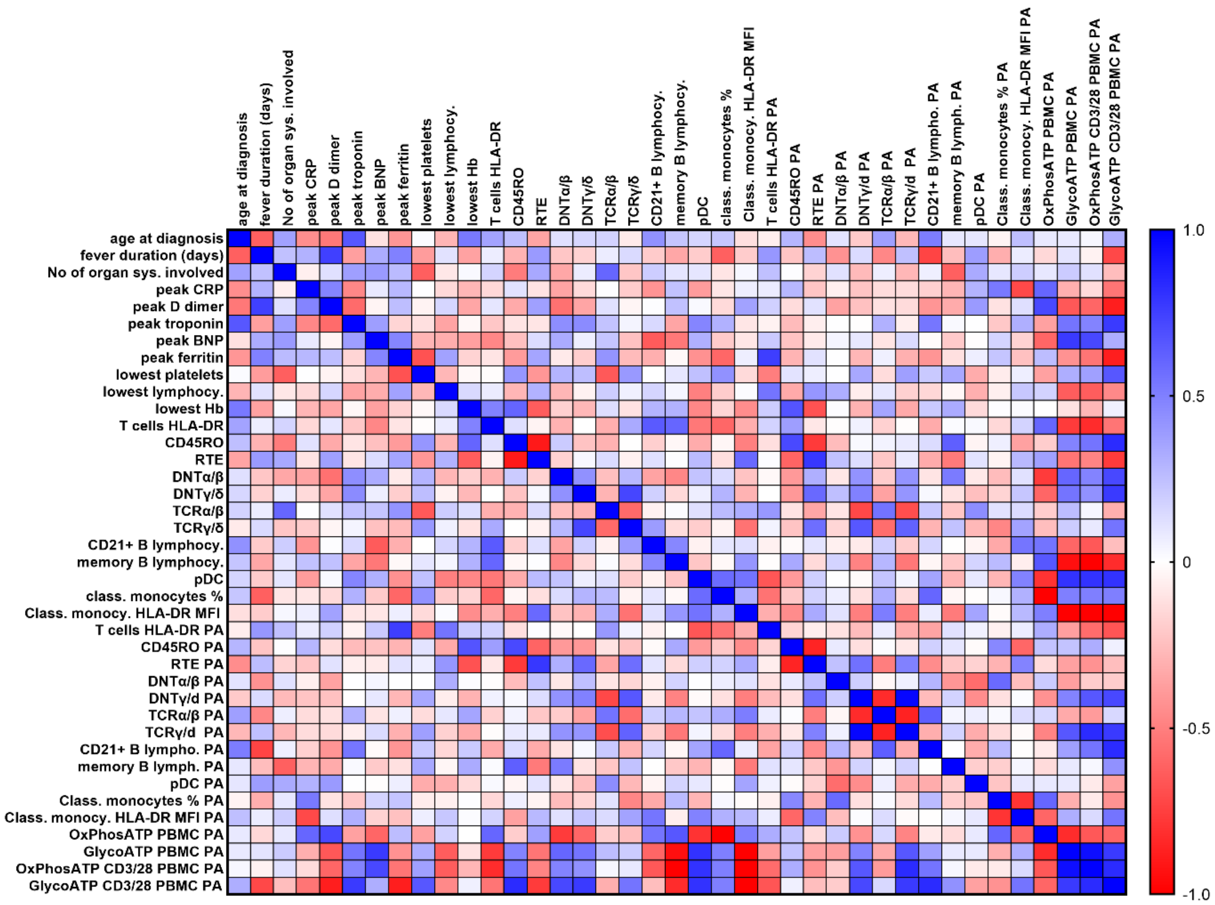

Figure S12: The association of clinical and immunological markers (Spearman correlation coefficient) between the selected clinical data, flow cytometry data and metabolic parameters in acute phase of MIS-C and in the convalescent phase (PA).
